# Using electronic medical records in hospital simulation for infection control intervention assessment

**DOI:** 10.1017/ice.2024.224

**Published:** 2025-03

**Authors:** Fardad Haghpanah, Eili Y Klein, for the CDC MInD-Healthcare Program

**Affiliations:** 1 One Health Trust, Washington, D.C., USA; 2 Department of Emergency Medicine, Johns Hopkins School of Medicine, Baltimore, MD, USA

## Abstract

**Background:**

Clinical trials for assessing the effects of infection prevention and control (IPC) interventions are expensive and have shown mixed results. Mathematical models can be relatively inexpensive tools for evaluating the potential of interventions. However, capturing nuances between institutions and in patient populations have adversely affected the power of computational models of nosocomial transmission.

**Methods:**

In this study, we present an agent-based model of ICUs in a tertiary care hospital, which directly uses data from the electronic medical records (EMR) to simulate pathogen transmission between patients, HCWs, and the environment. We demonstrate the application of our model to estimate the effects of IPC interventions at the local hospital level. Furthermore, we identify the most important sources of uncertainty, suggesting areas for prioritization in data collection.

**Results:**

Our model suggests that the stochasticity in ICU infections was mainly due to the uncertainties in admission prevalence, hand hygiene compliance/efficacy, and environmental disinfection efficacy. Analysis of interventions found that improving mean HCW compliance to hand hygiene protocols to 95% from 70%, mean terminal room disinfection efficacy to 95% from 50%, and reducing post-handwashing residual contamination down to 1% from 50%, could reduce infections by an average of 36%, 31%, and 26%, respectively.

**Conclusions:**

In-silico models of transmission coupled to EMR data can improve the assessment of IPC interventions. However, reducing the uncertainty of the estimated effectiveness requires collecting data on unknown or lesser known epidemiological and operational parameters of transmission, particularly admission prevalence, hand hygiene compliance/efficacy, and environmental disinfection efficacy.

## Introduction

Hospital-acquired infections (HAIs) pose a significant threat to patient safety and burden the US healthcare system with an excess $8 to $12 billion annually due to prolonged hospital stays and increased mortality risk.^1^ The mechanisms by which organisms spread between patients is complex and poorly understood, hampering assessments of intervention effectiveness. The primary pathways of transmission are presumed to include lapses in infection control practices by healthcare workers (HCWs) and contamination of medical equipment and the hospital environment.^2–4^ However, the complexity and heterogeneity of HCW-patient contact patterns and frequent contacts with surfaces and devices, many of which are untraceable,^5^ make it difficult to measure and evaluate the relative role of each pathway.

A valuable, yet underutilized, source of data to reduce the uncertainties in understanding of transmission pathways is electronic medical records (EMRs). EMR data contains time-stamped information on patient movements and interactions with HCWs. Prior studies have utilized EMR data to develop network models to assess transmission rates using statistical models.^6–8^ These studies found that the structure of the patient-nurse network can increase the risk of transmission. However, understanding the relative role of different transmission pathways (eg, environmental versus HCW-mediated) and the potential impact of interventions remains limited. A more robust modeling process is needed to effectively tie together information on patient location and the HCW-patient contact network with transmission and infection events derived from EMR data.

In this study, we present an agent-based simulation of the ICUs in the Johns Hopkins Hospital (JHH), hereafter referred to as *The Hospital*, coupled to JHH’s EMR data to simulate transmission of two HAI-causative pathogens: Methicillin-Resistant Staphylococcus Aureus (MRSA) and Vancomycin-Resistant Enterococci (VRE). We investigated the effects of parameter uncertainty on model outcomes to provide insight about the most important sources of randomness, for which further data collection could help reduce uncertainty. Using our model, we also simulated a number of interventions and estimated their impacts on reducing infections.

## Methods

With the high operational cost of randomized clinical trials for the assessment of infection control interventions, we advocate for simulation techniques as an alternative approach. The novelty of our simulation-based framework is using a hospital’s EMR data to tailor the simulations to the operations and environment of the hospital.

### Clinical data collection

#### Electronic medical records

All encounter and admission/discharge information for all patients that were hospitalized in the adult ICUs were extracted in bulk from The Hospital’s EMR system. The Hospital uses the Epic® Medical Record System for the entire enterprise. Patients’ and HCWs’ identification information were masked for privacy protection, according to the Institutional Review Board (IRB) Authorization Agreement. The de-identified data sets were downloaded onto a secure virtual desktop for sharing and analysis.

This study is focused on the six adult ICUs at The Hospital: Cardiac Care Unit (CCU), Cardiovascular Surgical ICU (CVSU), Medical Intensive Care Unit (MICU), Neuro Critical Care Unit (NCCU), Surgical ICU (SICU), and Weinberg Surgical ICU (WSICU).

Time-stamped data from laboratory results (including surveillance culture results), medication administration, and flowsheet information (which includes vital signs and other reported medical interventions), were used to identify HCW-patient interactions following the same methodology as Klein et al.^8^ Briefly, medication administration and laboratory specimen collection were assumed to represent an actual HCW interaction. Flowsheet data is more complex and includes auto-populated data; thus, an algorithm was used to filter events judged as likely to be automated events. As an HCW-patient interaction could consist of multiple events within a short period of time (eg, multiple medication administrations, specimen collections, or vital signs), we combined separate line-items from a single HCW that occurred within 15 minutes of each other and created an interaction start and end time.

#### Event queue construction

We compiled time-stamped HCW interactions with patient location data for the period of June 1, 2017, to July 1, 2018, and sorted all events by time to create an event queue data file. The event queue provides the time and location of patient-HCW interactions and movement of patients (admission, discharge, and transfer) to be simulated.

#### Simulations

We used Agent-Based Modeling (ABM) to simulate colonization and infection events due to the interactions between patients, HCWs, and room environments, in the ICUs of The Hospital.

ABM is a bottom-up modeling technique where complicated global behaviors of systems, or complex processes such as nosocomial transmission of HAIs, can be predicted by modeling the fundamental entities (agents) of the system/process and defining their interactions with each other and the environment.^9^ Each agent interacts with other agents and the environment based on a set of probabilistic and deterministic decision rules.

#### The agent-based model

Our agent-based model of HAI-causing pathogen transmission is based on our previous work on the study of the effects of uncertainties on the dynamics of nosocomial transmission of HAIs.^10^ The model consists of six ICUs and includes patients and HCWs modeled as agents (ie, separate entities). Below is a summary of the modeling specifications (details in Appendix A).

#### Model dynamics

##### Daily routine

On admission, a new patient is generated, unless in readmission cases where the corresponding patient is retrieved from the model memory. Each patient is assigned to their room, with their disease state assigned randomly based on the admission status probabilities. On discharge, the terminal disinfection protocol is executed in the patient’s room to clear the room contamination based on an efficacy rate ( 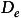 ).

For contact events, the following steps are simulated:

- Before a patient contact:
  - For patients under contact precautions, the HCW may comply with wearing PPE with some probability ( 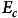 ). Otherwise, the HCW may comply to hand washing with a probability ( 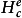 ), which can remove the contamination from the HCW’s hands, though the contamination on their clothes (eg, sleeves, scrubs, etc.) may remain ( 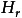 ).
  - The HCW may contact the room environment. This allows for both environmental shedding by contaminated HCWs ( 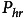 ) and HCW contamination from a contaminated room ( 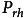 ).
- During a patient contact:
  - If the HCW or their PPE is contaminated, a susceptible patient may become colonized with some probability ( 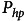 ).
  - Similarly, a colonized patient can contaminate the HCW or their PPE with some probability ( 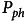 ).
- After a patient contact:
  - The HCW may contact the patient’s environment again, which with a probability may lead to environmental contamination if the HCW or their PPE is contaminated ( 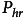 ).
  - The HCW discards their PPE, if using, otherwise, they may comply with hand washing with a probability ( 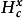 ) when exiting the room.

To account for direct environmental colonization from a contaminated room (aside from HCW-mediated environmental contamination), a Bernoulli trial is conducted every hour for patients with a success probability equal to the hourly probability of direct environmental colonization ( 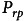 ).

Colonization screening is performed once every 7 days on a fraction of patients, determined by the screening compliance level ( 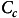 ) using a Bernoulli trial with a success probability equal to the test accuracy ( 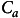 ).

The disease state of colonized patients may progress into infected with some probability at any time during their stay. This probability is estimated based on the data for the time of infection onset from admission. Time of infection onset was assumed to be the time of specimen collection for the infection diagnosis test. Based on this data, the probability distribution of time-to-infection ( 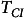 ) was constructed with a Gaussian Kernel Density function (Figure A1).

#### Parameterization

We defined the probability distribution of random variables in the model based on the findings from the literature (Table 1). We used uniform distributions to allow for an unbiased uncertainty analysis where there was no evidence in the literature suggesting more informative distributions. For parameters that were informed from the EMR data, we fitted Uniform, Beta, and Gaussian Kernel Density distributions.

**Table 1.** Model parameters and their probability distributions

**Parameter**	**Prior distribution** ^**(1)**^	**Source**	**Relaxed distribution** ^**(2)**^	**Sampled per**
Admission prevalence ( 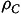 )	*U* [ 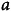 = 0.01, 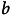 = 0.15]^(3)^	EMRs	*U* [ 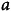 = 0, 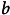 = 0.5]	Simulation
Culture test accuracy ( 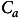 )	*U* [ 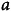 = 0.4, 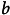 = 0.95]	^22,23^	*U* [ 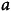 = 0, 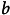 = 1]	Simulation
Active surveillance compliance ( 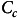 )	*U* [ 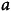 = 0.75, 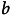 = 0.9]	EMRs	*U* [ 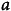 = 0.75, 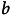 = 1]	Simulation
Mean hand hygiene compliance probability on entry ( 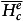 )	*U* [ 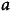 = 0.5, 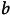 = 0.7]	^24–27^	*U* [ 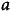 = 0.5, 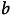 = 1]	HCW
Mean hand hygiene compliance probability on exit ( 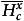 )	*U* [ 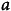 = 0.6, 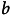 = 0.9]	^24–27^	*U* [ 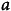 = 0.6, 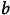 = 1]	HCW
Hand hygiene compliance variability from mean ( 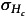 )	*U* [ 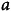 = 0, 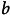 = 0.2]	Assumed	*U* [ 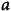 = 0, 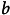 = 0.2]	Simulation
Hand hygiene compliance probability ( 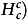	*Beta* ( 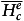 , 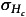 )^(4)^	Assumed	*Beta* ( 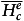 , 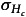 )	HCW
Mean HCW PPE compliance probability ( 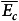 )	*U* [ 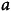 = 0.8, 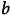 = 0.9]	^24,28^	*U* [ 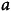 = 0.8, 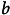 = 1]	Simulation
HCW PPE compliance variability from mean ( 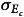 )	*U* [ 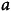 = 0, 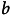 = 0.2]	Assumed	*U* [ 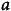 = 0, 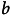 = 0.2]	Simulation
HCW PPE compliance probability ( 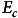 )	*Beta* ( 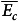 , 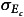 )	Assumed	*Beta* ( 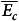 , 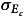 )	HCW
Residual contamination post-handwashing ( 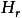 )	*U* [ 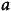 = 0, 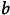 = 1]	Assumed	*U* [ 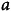 = 0, 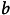 = 1]	Simulation
Terminal room disinfection efficacy ( 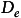 )	*U* [ 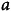 = 0.4, 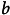 = 0.6]	^26^	*U* [ 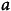 = 0.4, 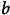 = 1]	Simulation
Probability of room contamination by colonized patients per day ( 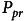 )	*U* [ 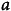 = 0.4, 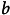 = 0.8]	^13^	*U* [ 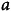 = 0, 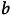 = 1]	Simulation
Probability of transmission from contaminated HCW to susceptible patient ( 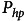 )	*U* [ 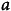 = 0, 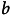 = 1]	Assumed	*U* [ 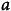 = 0, 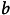 = 1]	Simulation
Probability of HCW contamination from a colonized patient ( 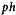 )	*U* [ 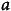 = 0.4, 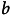 = 0.8]	^13^	*U* [ 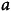 = 0, 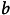 = 1]	Simulation
Probability of HCW contamination from contaminated environment ( 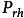 )	*U* [ 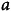 = 0.4, 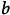 = 0.8]	^13^	*U* [ 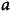 = 0, 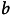 = 1]	Simulation
Probability of environmental contamination from contaminated HCW ( 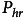 )	*U* [ 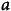 = 0.4, *b* = 0.8]	^13^	*U* [ 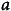 = 0, 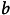 = 1]	Simulation
Progression time from colonization to infection ( 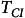 )	GKDE ( 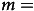 7.1 days, IQR: 1.2–33.4)^(5)^	EMRs	N/A	Patient
Probability of patient direct environmental colonization per hour ( 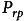 )	*U* [ 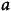 = 0, 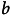 = 0.2]	Assumed	*U* [ 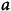 = 0, 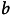 = 0.2]	Simulation
Shedding increase factor for infected ( 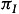 )	*U* [ 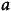 = 1, 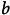 = 2]	Assumed^29^	*U* [ 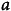 = 1, 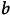 = 2]	Simulation
Pathogen natural clearance rate from dry surfaces per day ( 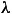 )	*U* [ 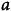 = 0, 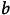 = 0.01]	^30,31^	*U* [ 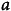 = 0, 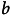 = 0.1]	Simulation

(1) Used for intervention simulations.

(2) Used for uncertainty analysis.

(3) *U[a, b]* denotes uniform distribution where *a* and *b* are the lower and upper bounds, respectively.

(4) *Beta(μ, σ)* denotes Beta distribution where *μ* and *σ* are the mean and standard deviation. The shape parameters of the Beta distribution can be obtained as follows: *α = (μ* ^*2*^ *– μ* ^*3*^ *– μσ* ^*2*^ *)/σ* ^*2*^ *and β = α(1/ μ – 1).*

(5) *GKDE(m, IQR)* denotes Gaussian Kernel Density where *m* and *IQR* are median and interquartile range, respectively.

The contamination-related parameters were assumed to take equal values as there is no evidence that would suggest otherwise. All the other parameters were assumed to be independent due to lack of correlation evidence in the literature.

#### Parameter identifiability

In the absence of data, we defined the following identifiability scenarios for probability of environmental direct colonization (*P*_*rp*_) and post-handwashing residual contamination (*H*_*r*_) as unknown parameters (Table 2): Low and High Environmental colonization (denoted by LE and HE, respectfully), and Low and High Residual contamination (denoted by LR and HR, respectfully).

**Table 2.** Identifiability scenarios: **LELR** denotes low environmental colonization and low residual contamination; **LEHR** denotes low environmental colonization and high residual contamination; **HELR** denotes high environmental colonization and low residual contamination; and **HEHR** denotes high environmental colonization and high residual contamination

Scenario name	Post-handwashing residual contamination	Patient direct environmental colonization rate	Other parameters
LELR	U [ 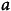 = 0.0, 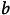 = 0.1]	U [ 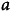 = 0.00, 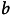 = 0.01]	See Table 1
LEHR	U [ 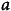 = 0.1, 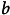 = 1]	U [ 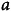 = 0.00, 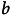 = 0.01]	See Table 1
HELR	U [ 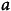 = 0.0, 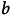 = 0.1]	U [ 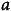 = 0.10, 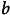 = 0.15]	See Table 1
HEHR	U [ 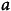 = 0.1, 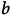 = 1]	U [ 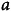 = 0.10, 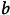 = 0.15]	See Table 1

### Uncertainty analysis

We used partial rank correlation coefficients (PRCCs) to investigate the effects of parameter uncertainty on model output. For uncertainty analysis, the simulations were repeated while relaxing parameter distributions (see Table 1).

We also investigated the effects (sensitivity) of parameter distribution boundaries on PRCC calculations. For doing so, we changed the upper and lower bounds of the distribution of each parameter and repeated the PRCC analysis.

## Results

### Summary statistics of EMRs

There were variations in capacity, admission rate, length of stay (LOS), HCW contact patterns, and infection rate across the ICUs (see Tables B1 and B2 and Figure B1). The medium number of visits each patient received in an hour was lowest in the NCCU (0.8) and highest in the SICU (2.2). Most contacts were by registered nurses (69%–83%). The remainder of the contacts involved more than 40 other types of HCWs, most commonly technicians, respiratory therapists, and nurse practitioners.

### Model calibration results

The model parameters could only be fitted to the observed number of infections when post-handwashing residual contamination was assumed to take values smaller than 10%, that is, under the LELR (low environmental colonization and low residual contamination) and HELR (high environmental colonization and low residual contamination) scenarios (Figure B2). This implies that post-handwashing residual contamination is unlikely to take large values as the model could not reproduce the observed infection rates under such an assumption.

### Results of uncertainty analysis

#### Partial rank correlation coefficients

The results from our uncertainty analysis revealed that the two major sources of uncertainty in determining the rate of infection were admission prevalence and hand hygiene compliance levels. Other parameters also showed moderate to significant contributions to infection uncertainty depending on the parameter identifiability scenario (Table 3).

**Table 3.** Parameters with above-moderate correlation coefficients (defined as partial rank correlation coefficients larger than 0.3 for positive correlation and smaller than −0.3 for negative correlation) under different parameter identifiability scenarios

**Identifiability scenario: LELR** (low environmental colonization and low residual contamination)	
Admission prevalence	0.97***
residual contamination post-handwashing	0.48***
Mean hand hygiene compliance probability on entry	−0.42***
Mean hand hygiene compliance probability on exit	−0.35**
Terminal room disinfection efficacy	−0.30***
**Identifiability scenario: LEHR** (low environmental colonization and high residual contamination)	
Admission prevalence	0.94***
Terminal room disinfection efficacy	−0.32***
**Identifiability scenario: HELR** (high environmental colonization and low residual contamination)	
Admission prevalence	0.96***
Mean hand hygiene compliance probability on entry	−0.61***
Mean hand hygiene compliance probability on exit	−0.52***
Residual contamination post-handwashing	0.40***
Probability of environmental contamination from contaminated HCW	0.36***
**Identifiability scenario: HEHR** (high environmental colonization and high residual contamination)	
Admission prevalence	0.92***
Terminal room disinfection efficacy	−0.39***

(***) Significant at the 1% level; (**) Significant at the 5% level.

The parameters that were correlated with infections also varied across the ICUs (Tables B3-B6). For example, mean hand hygiene compliance on exit showed a weak correlation with infections in the CVSU and NCCU while showed stronger correlations in the other four ICUs. Also, the probability of environmental contamination from HCWs was moderately correlated with infections in the MICU, while it showed weak correlations in the CVSU and NCCU.

The results also suggested nonlinear correlations between infections and certain model parameters (Table 4). For example, the mean hand hygiene compliance level was only correlated with infections when compliance varied above 50%. That is, changes in mean compliance level from 0 to 50% did not reduce infections.

**Table 4.** Parameters with statistically significant sensitive partial rank correlation coefficients (PRCCs) to distribution boundaries (from the half-range^(1)^ uncertainty analysis)

**Parameter**	**Lower distribution range** **(PRCC)**	**Upper distribution range** **(PRCC)**
Probability of HCW contamination from contaminated environment	0 – 50% ( 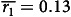 )	50 – 100% ( 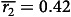 )
Mean hand hygiene compliance on entry	50 – 75% ( 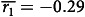 )	75 – 100% ( 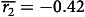 )
Mean hand hygiene compliance on exit	50 – 75% ( 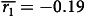 )	75 – 100% ( 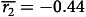 )
Hand hygiene compliance variability from mean	0 – 10% ( 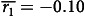 )	10 – 100% ( 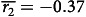 )
Terminal room disinfection efficacy	40 – 70% ( 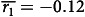 )	70 – 100% ( 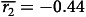 )
Probability of environmental contamination from contaminated HCW	0 – 50% ( 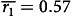 )	50 – 100% ( 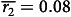 )

(1) If a parameter takes values between *a* and *b* (ie, [*a – b*]), with *m* as the middle point, lower and upper half-ranges are defined as [*a – m*] and [*m – b*], respectively.

The details of the uncertainty analysis for each ICU are elaborated in Appendix B.

#### Intervention selection

Based on the definition of statistical correlation, parameters with strong correlation coefficients (PRCCs) with infections can be regarded as potentially more effective interventions. Thus, the following interventions were defined to be simulated: improving hand hygiene compliance among HCWs (improving mean compliance among all HCWs to 95% and improving compliance among the least-compliant HCWs to 75% to reduce variation from mean), hand hygiene efficacy (through reducing mean post-handwashing residual contamination from 50% to 1%), and improving mean terminal room disinfection efficacy from 50% to 95%. As admission prevalence cannot be reduced through an intervention in a single hospital, we excluded lowered admission prevalence as an intervention to be simulated.

### Results of intervention simulations

**Figure 1.**
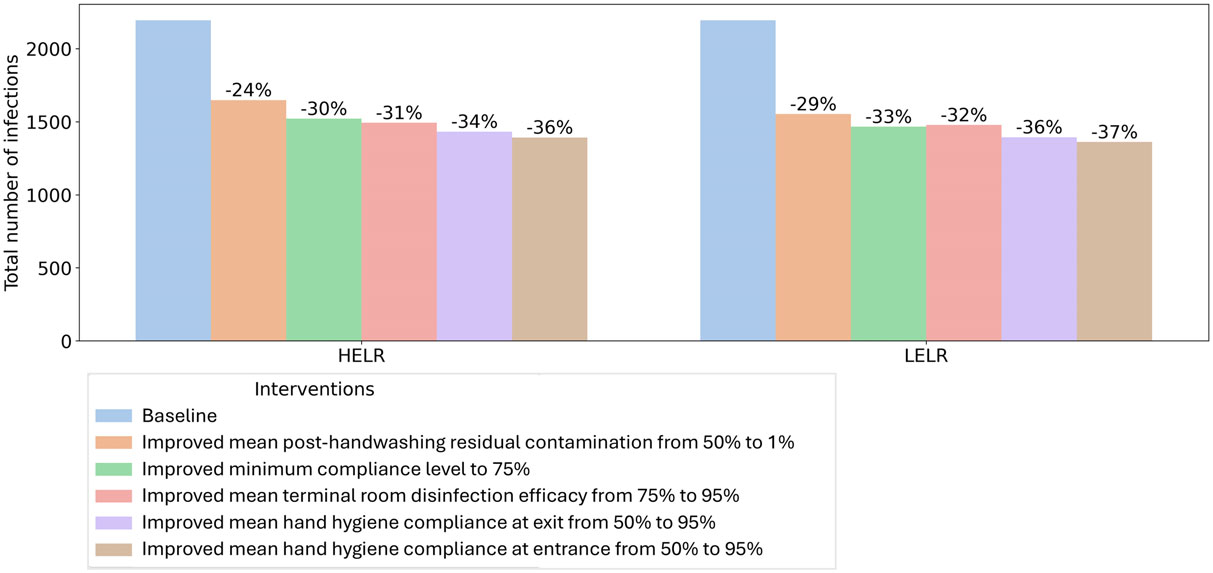
Results of intervention simulations under different parameter scenarios. Bar values show the average impact of each intervention on reducing infections. **LELR**: low probability of direct environmental colonization and low levels of residual contamination; **HELR**: high probability of direct environmental colonization and low levels of residual contamination.

Although all the selected interventions showed comparable impacts on reducing infections, improving the mean handwashing compliance to 95% had the largest impact, resulting in an average of 36% reduction in total number of infections in the hospital (Figure 1). The order of interventions in terms of impact on reducing infections are (from larger to smaller): improving the mean terminal room disinfection efficacy to 95% (average impact: 31%), improving minimum hand hygiene compliance to 75% (average impact: 31%), and reducing mean post-handwashing residual contamination to 1% (average impact: 26%).

**Figure 2.**
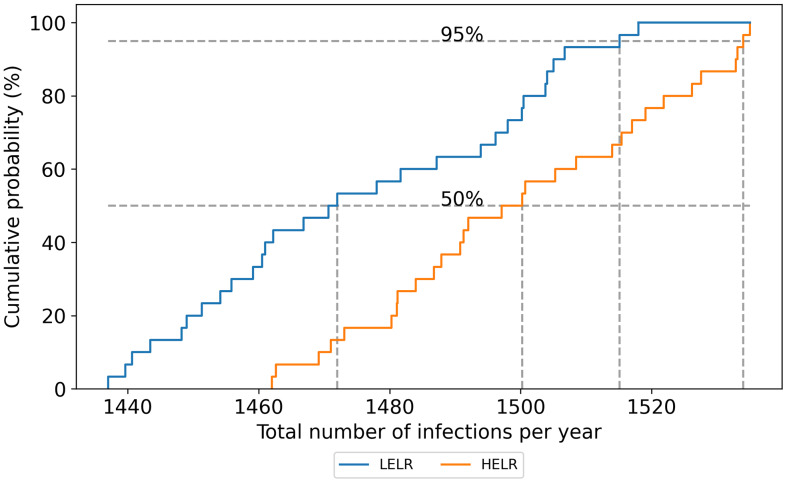
Cumulative probability plot for the annual number of infections after improving terminal room disinfection efficacy to 95% through an intervention. Dashed lines show the 50^th^ (median) and 95^th^ percentiles. **LELR**: low probability of direct environmental colonization and low levels of residual contamination; **HELR**: high probability of direct environmental colonization and low levels of residual contamination.

Our probabilistic methodology provides the uncertainty level in the estimation of the impact of interventions, as well. This explains the certainty level of the estimated impact of interventions due to all sources of randomness or unknowns. For example, improving the mean terminal room disinfection efficacy to 95% had an average impact of 31% in reducing infections. That is from 2192 cases in baseline to at least 1478 cases with a 50% probability. However, the cumulative probability plot of the intervention impact (Figure 2) shows that the infections would be reduced to at least 1505 cases with a probability of 95%. See Appendix B: Results, Section B3.4 for the probabilistic plots of the rest of the interventions.

## Discussion

Our results support the claim that more data on infection control and environmental contamination is needed to better estimate the potential impact of interventions. Meanwhile, we also demonstrate that there are still significant gains to be made from exploiting already available data. In particular, EMR data can address some of the uncertainties in the behavior of HCWs and patients. Here, we demonstrated their utility to evaluate the potential impact of interventions to reduce transmission using simulation. Importantly, our methods are generalizable by tying intervention evaluations directly to data extracted from the EMR of a hospital, allowing hospitals to tailor intervention simulations to their own operations and environment.

Although our results may not be applicable to all hospitals, as intervention effectiveness may differ across hospitals, our model and results point to some important general data gaps. Firstly, parameterization issues were most pronounced in the environmental components of our model. This accords with the lack of literature regarding pathogen transfer between different surfaces, patients, and HCWs. Although some studies have shown the potentially significant role of the environment in transmission,^11–13^ challenges remain in accurate quantification of the role of the hospital environment in transmission.

Second, our uncertainty analysis identified admission prevalence as the most critical model parameter. Increasing levels of pathogen prevalence at admission increases colonization pressure, which in turn, increases risk of transmission.^14^ Inaccurate estimation of admission prevalence risks overestimation of transmission parameters, leading to an incorrect estimation of the impact of interventions. More extensive admission testing is likely required to address the uncertainty in admission prevalence in ICUs.

Third, we found that interventions for improving hand hygiene compliance is complicated by the uncertainty in the level of compliance among HCWs. In accord with the literature,^15,16^ we found that increasing the mean handwashing compliance was highly impactful. However, our simulations also suggest that reducing the variability among the HCWs, or more specifically, improving compliance among the least compliant HCWs, was comparably effective. Further studies are needed to assess the cost-effectiveness of improving overall compliance or retraining low-compliant HCWs to a pre-established minimum threshold.

Fourth, we found a strong degree of sensitivity to post-handwashing residual contamination. Although interventions have largely been targeted at handwashing compliance^16^ and improved efficacy in removing contamination from HCWs’ hands,^17^ studies have also shown that long-sleeved white coats and ties are frequently contaminated with HAI-causing pathogens.^18,19^ Thus, in recent years, a number of healthcare facilities in the UK and US have instituted “bare below the elbow” mandates (BBE) to reduce the likelihood of post-handwashing contamination. BBE typically not only bans neckties and long sleeves, but also non-medical devices (eg, cellphones) or cosmetic accessories (eg, jewelry).^20^ Our results suggest that reducing post-handwashing contamination from all these potential sources of contamination should be a focus of future research, particularly, we need more clinical studies and data collection to better quantify the role of post-handwashing contamination in transmission, as currently this theoretically important parameter is completely unknown.

In a nutshell, for more effective intervention assessment through modeling, future research must prioritize areas with the greatest uncertainty. For example, the role of environmental contamination along with HCW-mediated transmission (ie, patient-to-HCW-to-patient) is critical to be understood as each requires different interventions to mitigate transmission. For example, when environmental colonization was assumed low, our model suggested that improving hand hygiene was more effective than scenarios in which environmental colonization was assumed high. Collecting measures of pathogen absolute abundance (ie, pathogen load) on patient room surfaces combined with HCW visit and patient movement data from EMR, could provide a rich tapestry describing the patient-environment-HCW network and the role of patient and HCW shedding in transmission.

## Challenges and limitations

First, while EMRs provide a rich source of data on patient and HCW movements and encounters, not all HCW-patient interactions are documented. Second, certain structural assumptions had to be made due to lack of data (eg, HCW-environment contacts in patient rooms) and certain behaviors had to be excluded, for example, HCW-to-HCW contamination, HCW contamination outside of patient rooms, and environmental contamination by visitors and custodians, as none of these activities is recorded in EMRs. Third, we did not account for patient-specific risk factors of susceptibility to colonization due to lack of sufficient data. The decision to exclude many of these details originates from either lack of data or insufficient knowledge about their epidemiological mechanisms of increasing risk of colonization. For example, antibiotic exposure was found to increase risk of acquisition from 20% to 90%.^8,21^ Given the uncertainty in the impact of antibiotics, from a statistical analysis perspective, one may infer that there might be other confounding parameters that are needed to be included to correctly evaluate the role of antibiotic exposure as a colonization risk factor for different patients.

Lastly, the computational cost of our simulations is extremely high, limiting the number of realizations we could obtain. Future work is needed to develop more computationally tractable simulations, particularly for parameter estimation. Advancements in neural networks may be a means to improve parameter estimation in such complex simulations.

## Supporting information

Haghpanah et al. supplementary material 1 Haghpanah et al. supplementary material

Haghpanah et al. supplementary material 2 Haghpanah et al. supplementary material

## Data Availability

This study was approved by the Johns Hopkins Institutional Review Board. The electronic medical records used in this study was acquired from the Johns Hopkins Hospital’s EMR system. Due to patient privacy, the data used in this study is not available for public sharing.

## Supplementary material

The supplementary material for this article can be found at https://doi.org/10.1017/ice.2024.224

## Code availability

The code used to conduct the simulations and generate figures is available in the following GitHub repository: https://github.com/fardadhp/nosocomial_infections_ABM.
