# Supplementary material for: Using electronic medical records in hospital simulation for infection control intervention assessment
Source: Infect Control Hosp Epidemiol. 2025 Jan 9;46(3):298–304. doi: 10.1017/ice.2024.224 (PMC11883657; doi:10.1017/ice.2024.224)
Supplement: Haghpanah et al. supplementary material 1 — Haghpanah et al. supplementary material [file S0899823X24002241sup001.docx]

**Supplementary Materials for:**

Using electronic medical records in hospital simulation for infection control intervention assessment

Fardad Haghpanah^1*^ and Eili Klein^1,2^; for the CDC MInD-Healthcare Program

^1^ One Health Trust, Washington, D.C., USA

^2^ Department of Emergency Medicine, Johns Hopkins School of Medicine, Baltimore, MD, USA

^*^ Corresponding author:

5636 Connecticut Avenue NW,
PO Box 42735, Washington, D.C.
20015, U.S.A.

[haghpanah@onehealthtrust.org](mailto:haghpanah@onehealthtrust.org)

**Appendix A: Methods**

**A2.2. Simulations**

*A2.2.1. The agent-based model*

Agent-Based Modeling (ABM) is a bottom-up simulation technique where complicated global patterns in a system can be simulated by modeling the fundamental elements (agents) of the system and their interactions with each other and the environment [1]. The agent interactions are based on a set of decision rules while accounting for the underlying heterogeneities and uncertainties. Woven together with EMR data, the ABM framework offers a means to conduct a micro-scale heterogeneous investigation of the underlying mechanisms of HAI transmission and their associated uncertainties. These uncertainty analyses can help to identify the most important sources of uncertainty, hence, improving our understanding of transmission pathways.

*A2.2.2. Model dynamics*

A2.2.2.1. Simulation setup

At the beginning of each simulation, the hospital is initialized by creating the selected ICU departments. The hospital-wide event queue data is loaded, and the parameters of the model are adjusted if an intervention scenario is set to be implemented.

A2.2.2.2. Daily routine

The activities in each ICU are divided into two groups: (1) those with known time of occurrence from the event queue, and (2) activities with unknown time of occurrence, which are set to occur at the start of each day. These activities include colonized patients developing an infection based on probability of progression ($P_{CI}$) and pathogen clearance from rooms environment based on the daily rate of natural clearance ($\lambda$).

The time-stamped activities from the event queue are processed in order. On admission, a new patient is generated, unless in readmission cases where the corresponding patient agent is retrieved from the model memory. Each admitted/readmitted patient is assigned to their room, with their disease state determined from a multinomial trial using the admission status probabilities. The disease state of readmitted patients is assumed to be unchanged since their last discharge.

On discharge, the terminal disinfection protocol is executed in the patient’s room, which can clear the room contamination based on the room disinfection efficacy level ($D_{e}$). In the case of transfers (i.e., when a patient is readmitted after discharged), the patient agent is not deleted from memory.

For contact events, the following steps are simulated:

- Before patient contact:
  - The HCW may comply with wearing PPE with some probability ($E_{c}$) if the patient is under contact precautions.
  - If the patient is not under contact precautions, the HCW may wash their hands given the HCW hand hygiene compliance probability on entry ($H_{c}^{e}$), which can remove the contamination from the HCW hands, though the contamination on their clothes (e.g., sleeves, scrubs, etc.) may remain based on the post-hand-washing residual contamination probability ($H_{r}$).
  - The HCW may contact the environment of the patient’s room. This allows for both environmental shedding by contaminated HCWs ($P_{hr}$) and HCW contamination from a contaminated room ($P_{rh}$).
- During patient contact:
  - If the HCW or their PPE is contaminated, the patient may become colonized with some probability ($P_{hp}$).
  - Similarly, a colonized patient can contaminate the HCW or their PPE with some probability ($P_{ph}$).
- After patient contact:
  - The HCW may contact the patient’s environment again, which with a probability may lead to environmental contamination if the HCW or their PPE is contaminated ($P_{hr}$).
  - The HCW discards their PPE, if using, otherwise, they may comply with hand washing when exiting the room, based on the hygiene compliance probability on exit ($H_{c}^{x}$), which could be different than that on entry (Table A1).

It is important to note that we assume that HCWs always correctly use PPE (when comply), and this does not affect the contamination state of HCWs. When a contaminated HCW uses PPE, she does not directly transmit pathogens to patients, unless the PPE was contaminated when the HCW contacted a patient’s environment before attending to the patient. However, since HCWs discard PPE after each visit, the risk of transmission to the next patient will remain unchanged. Furthermore, when a contaminated HCW uses PPE, it prevents environmental shedding from the HCW.

To account for direct environmental colonization from a contaminated room (aside from HCW-mediated environmental contamination), a Bernoulli trial is conducted every hour with a success probability equal to the hourly probability of direct environmental colonization ($P_{rp}$).

A2.2.2.3. Active surveillance

Per the infection prevention protocols in place at the time in the Hospital, patients were supposed to receive MRSA and VRE colonization screening within 48 hours of ICU admission, followed by regular screening every seven days afterwards. However, compliance rates with screening are not 100% due to several factors (e.g., workload, patient disposition, etc.). The screening compliance level during the study period (Jully 1, 2017, to July 1, 2018) was 78-88% for VRE and 76-90% for MRSA across the six ICUs (Table A1). In the model, we assume that colonization screening is performed once every 7 days on a fraction of patients, determined by the screening compliance level ($C_{c}$), starting from day 1, using a Bernoulli trial with a success probability equal to the test accuracy ($C_{a}$). For patients with a positive culture test, their status is changed to detected colonized (*DC*), and they are put under contact precautions for the rest of their stay.

To calculate compliance, we calculated the time between admission and first culture test. Those patients that had a culture test within 48 hours from admission were regarded as *screened on admission*. Patients that were transferred to other ICUs, or discharged and readmitted to the same ICU were regarded as new “admissions” and required to be screened again. The admission screening compliance was calculated by taking the ratio of *screened on admission* patients to all patients for each ICU.

Table A1. Admission surveillance compliance for VRE and MRSA during the study period

| **ICU** | **VRE surveillance compliance** | **MRSA surveillance compliance** |
| --- | --- | --- |
| CCU | 80% | 76% |
| CVSU | 80% | 80% |
| MICU | 88% | 90% |
| NCCU | 80% | 80% |
| SICU | 79% | 79% |
| WSICU | 78% | 80% |

A2.2.2.4. Infection from colonization

The disease state of colonized patients may progress into infected with some probability. This probability is estimated based on the distribution for the time from first positive culture test to time of infection onset. For each infected patient, the specimen collection time of the first positive culture test was taken as an estimation for time of colonization. For infected patients without a culture test, the admission time was used. Time of infection onset was assumed to be the time of specimen collection for the infection diagnosis test. The time difference between the two data entries was calculated as the *time-to-infection* variable (in hours). Based on this data, the probability distribution of time-to-infection ($T_{CI}$) was constructed with a Gaussian Kernel Density (Figure A1) using the scikit-learn package in python [2].

For each colonized patient, a sample is drawn from the time-to-infection distribution. If the patient’s time-to-infection ($\Delta t_{I}$) is shorter than the remaining time to discharge, the patient’s disease state is changed to *infected* (*I*) after $\Delta t_{I}$ hours. Infected patients are put under contact precautions for the remainder of their stay.


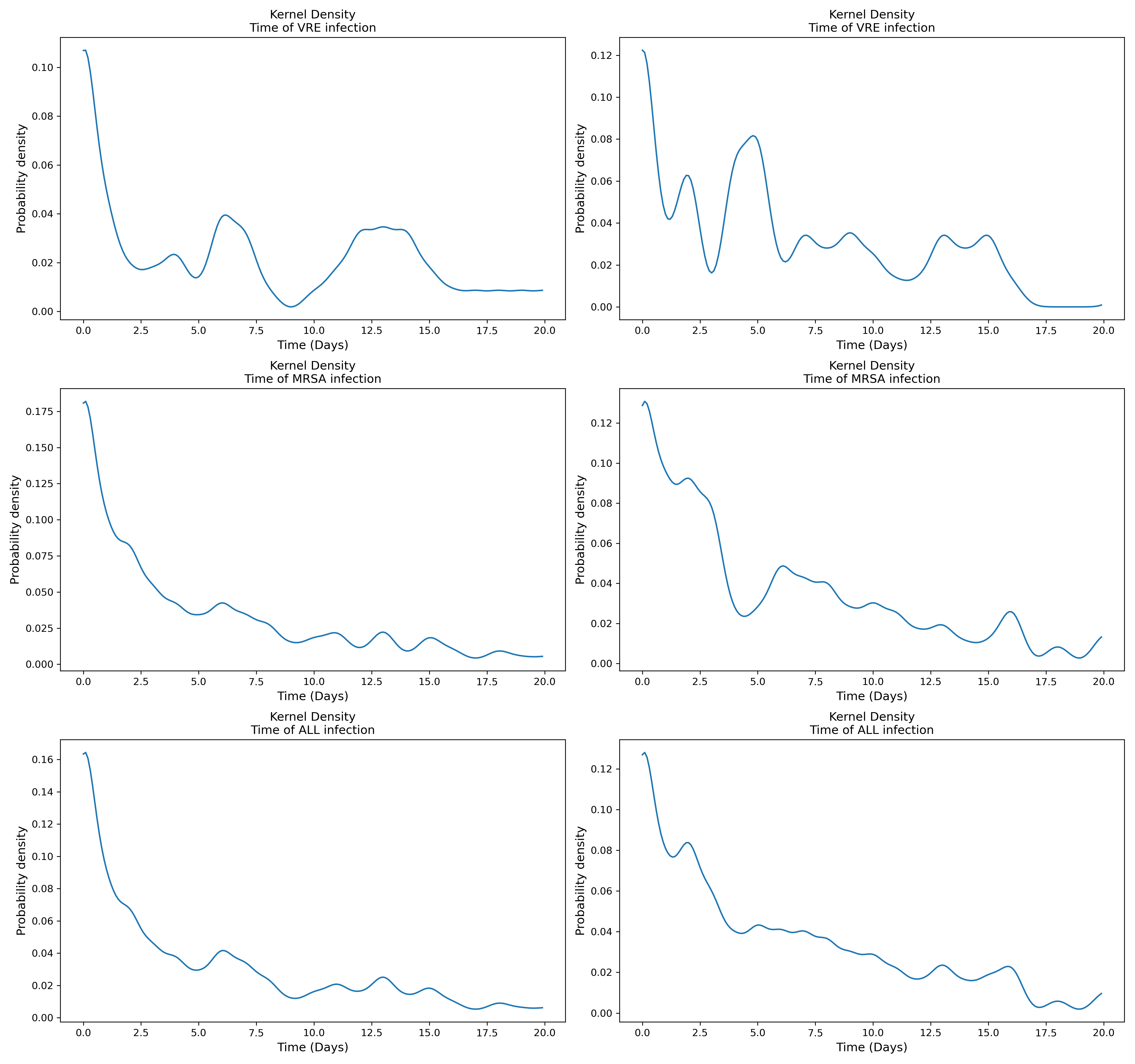


Figure A1. Gaussian kernel density estimation of time to infection from admission (left) and first positive culture test (right).

*A2.2.3. Parameterization*

A2.2.3.1. Prior distributions

We defined the probability distribution of random variables in the model based on the findings from the literature (Table A2). We used uniform distributions to allow for an unbiased uncertainty analysis.

The contamination-related parameters were assumed to take equal values as there is no evidence that would suggest otherwise. These parameters are the daily probability of room contamination by colonized patients ($P_{pr}$), the probability of HCW contamination by a colonized patient ($P_{ph}$), the probability of HCW contamination from contaminated environment ($P_{rh}$), and the probability of environmental contamination from a contaminated HCW ($P_{hr}$).

Table A2. Model parameters and their probability distributions

| **Parameter** | **Prior distribution^*^** | **Source** | **Relaxed distribution^**^** | **Sampled per** |
| --- | --- | --- | --- | --- |
| Admission prevalence ($\rho_{C}$) | *U* [$a$ = 0.01, $b$ = 0.15]^a^ | EMRs and [3,4] | *U* [$a$ = 0, $b$ = 0.5] | Simulation |
| Culture test accuracy ($C_{a}$) | *U* [$a$ = 0.4, $b$ = 0.95] | [5,6] | *U* [$a$ = 0, $b$ = 1] | Simulation |
| Active surveillance compliance ($C_{c}$) | *U* [$a$ = 0.75, $b$ = 0.9] | EMRs | *U* [$a$ = 0.75, $b$ = 1] | Simulation |
| Mean HCW hygiene compliance probability on entry ($\bar{H_{c}^{e}}$) | *U* [$a$ = 0.5, $b$ = 0.7] | [7–10] | *U* [$a$ = 0.5, $b$ = 1] | HCW |
| Mean HCW hygiene compliance probability on exit ($\bar{H_{c}^{x}}$) | *U* [$a$ = 0.6, $b$ = 0.9] | [7–10] | *U* [$a$ = 0.6, $b$ = 1] | HCW |
| HCW hygiene compliance variability from mean ($\sigma_{H_{c}}$) | *U* [$a$ = 0, $b$ = 0.2] | Assumed | *U* [$a$ = 0, $b$ = 0.2] | Simulation |
| HCW hygiene compliance probability ($H_{c}^{c})$ | *Beta* ($\bar{H_{c}^{e}}$, $\sigma_{H_{c}}$)^b^ | Assumed | *Beta* ($\bar{H_{c}^{e}}$, $\sigma_{H_{c}}$) | HCW |
| Mean HCW PPE compliance probability ($\bar{E_{c}}$) | *U* [$a$ = 0.8, $b$ = 0.9] | [7,11] | *U* [$a$ = 0.8, $b$ = 1] | Simulation |
| HCW PPE compliance variability from mean ($\sigma_{E_{c}}$) | *U* [$a$ = 0, $b$ = 0.2] | Assumed | *U* [$a$ = 0, $b$ = 0.2] | Simulation |
| HCW PPE compliance probability ($E_{c}$) | *Beta* ($\bar{E_{c}}$, $\sigma_{E_{c}}$) | Assumed | *Beta* ($\bar{E_{c}}$, $\sigma_{E_{c}}$) | HCW |
| Residual contamination post hand washing ($H_{r}$) | *U* [$a$ = 0, $b$ = 1] | Assumed | *U* [$a$ = 0, $b$ = 1] | Simulation |
| Terminal room disinfection efficacy ($D_{e}$) | *U* [$a$ = 0.4, $b$ = 0.6] | [9] | *U* [$a$ = 0.4, $b$ = 1] | Simulation |
| Probability of room contamination by colonized patients per day ($P_{pr}$) | *U* [$a$ = 0.4, $b$ = 0.8] | [12] | *U* [$a$ = 0, $b$ = 1] | Simulation |
| Probability of transmission from contaminated HCW to susceptible patient ($P_{hp}$) | *U* [$a$ = 0, $b$ = 1] | Assumed | *U* [$a$ = 0, $b$ = 1] | Simulation |
| Probability of HCW contamination from a colonized patient ($P_{ph}$) | *U* [$a$ = 0.4, $b$ = 0.8] | [12] | *U* [$a$ = 0, $b$ = 1] | Simulation |
| Probability of HCW contamination from contaminated environment ($P_{rh}$) | *U* [$a$ = 0.4, $b$ = 0.8] | [12] | *U* [$a$ = 0, $b$ = 1] | Simulation |
| Probability of environmental contamination from contaminated HCW ($P_{hr}$) | *U* [$a$ = 0.4, $b$ = 0.8] | [12] | *U* [$a$ = 0, $b$ = 1] | Simulation |
| Progression time from colonization to infection ($T_{CI}$) | GKDE ($m=$ 7.1 days, IQR: 1.2–33.4)^c^ | EMRs | N/A | Patient |
| Probability of patient direct environmental colonization per hour ($P_{rp}$) | *U* [$a$ = 0, $b$ = 0.2] | Assumed | *U* [$a$ = 0, $b$ = 0.2] | Simulation |
| Shedding increase factor for infected ($\pi_{I}$) | *U* [$a$ = 1, $b$ = 2] | Assumed [13] | *U* [$a$ = 1, $b$ = 2] | Simulation |
| Pathogen natural clearance rate from dry surfaces per day ($\lambda$) | *U* [$a$ = 0, $b$ = 0.01] | [14,15] | *U* [$a$ = 0, $b$ = 0.1] | Simulation |

^*^ Used for intervention simulations.

^**^ Used for uncertainty analysis.

^a^ *U[a, b]* denotes uniform distribution where *a* and *b* are the lower and upper bounds, respectively.

^b^ *Beta(μ, σ)* denotes Beta distribution where *μ* and *σ* are the mean and standard deviation. The shape parameters of the Beta distribution can be obtained as follows: *α = (μ^2^ – μ^3^ – μσ^2^) / σ^2^ and β = α(1/ μ – 1).*

^c^ *GKDE(m, IQR)* denotes Gaussian Kernel Density where *m* and *IQR* are median and interquartile range, respectively.

We estimated admission prevalence ($\rho_{C}$) in the ICUs during the study period from the EMR data by counting patients with a positive MRSA and/or VRE test within 48 hours from their admission times. While all patients were under culture surveillance during the study period, there could be missing importation cases due to the test accuracy and screening failure. Assuming an average of 70% test accuracy ($C_{a}=0.7$) for MRSA and VRE [5,6] and given the calculated screening compliance levels ($C_{c}$) in each ICU, the estimated admission prevalence ratios were adjusted for screening sensitivity (defined as the compound effects of accuracy and compliance) as $\rho_{C}^{adjusted}=\rho_{C}/(C_{a}C_{c})$. The estimated overall prevalence ratios (Table A2) in the six ICUs agree with the observed values in ICUs in the US for MRSA [3] and VRE [4], except for the MICU, where VRE admission prevalence was particularly high.

The event probabilities for the multinomial distribution of admission status were established based on the colonization importation ratio, i.e., admission prevalence ($\rho_{C}$). After sampling $\rho_{C}$ from its respective distribution, the probability of admission as susceptible, assuming no infection importation ($\rho_{I}=0$), can be calculated as: $\rho_{S}=1-\rho_{C}$. Given all event probabilities, the admission status of each patient is determined using a Multinomial trial, defined as *Multinomial (1, {*$\rho_{S}$*,* $\rho_{C}$*,* $\rho_{I}$*})*.

All the other parameters were assumed to be independent due to lack of correlation evidence in the literature.

Table A3. Estimated admission prevalence from the EMR data for the entire study period

| **ICU** | **MRSA** | | **VRE** | |
| --- | --- | --- | --- | --- |
|  | $\boldsymbol{\rho}_{\boldsymbol{C}}$ | **Adjusted** $\boldsymbol{\rho}_{\boldsymbol{C}}$ | $\boldsymbol{\rho}_{\boldsymbol{C}}$ | **Adjusted** $\boldsymbol{\rho}_{\boldsymbol{C}}$ |
| CCU | 4.3% | 8.1% | 1.1% | 2.0% |
| CVSU | 1.4% | 2.5% | 2.0% | 3.6% |
| MICU | 13.1% | 20.7% | 21.0% | 33.9% |
| NCCU | 2.5% | 4.4% | 1.9% | 3.4% |
| SICU | 3.6% | 6.5% | 5.4% | 9.8% |
| WSICU | 3.4% | 6.1% | 3.4% | 6.2% |

A2.2.3.2. Parameter identifiability

Parameter identifiability is a critical problem in complex biological models,[16] particularly agent-based models.[17] While we tried to inform our model with evidence-based parameter distribution boundaries, there still exist multiple completely or partially unknown parameters that could lead to model nonidentifiability. Three parameters are completely unknown: post-hand-washing residual contamination (including not only residual contamination on the HCW hands, but also clothes or other equipment; Hr), probability of transmission from contaminated HCW to susceptible patient (Php), and probability of patient direct colonization from the environment (Prp). In the absence of data, we defined the following identifiability scenarios for Prp and Hr (Table A4): Low and High Environmental colonization (denoted by LE and HE, respectfully), and Low and High Residual contamination (denoted by LR and HR, respectfully).

Table A4. Identifiability scenarios: **LELR** denotes low environmental colonization and low residual contamination; **LEHR** denotes low environmental colonization and high residual contamination; **HELR** denotes high environmental colonization and low residual contamination; and **HEHR** denotes high environmental colonization and high residual contamination.

| Scenario name | $H_{r}$ | $P_{rp}$ | Other parameters |
| --- | --- | --- | --- |
| LELR | U [$a$ = 0.0, $b$ = 0.1] | U [$a$ = 0.00, $b$ = 0.01] | See Table A2 |
| LEHR | U [$a$ = 0.9, $b$ = 1] | U [$a$ = 0.00, $b$ = 0.01] | See Table A2 |
| HELR | U [$a$ = 0.0, $b$ = 0.1] | U [$a$ = 0.10, $b$ = 0.15] | See Table A2 |
| HEHR | U [$a$ = 0.9, $b$ = 1] | U [$a$ = 0.10, $b$ = 0.15] | See Table A2 |

*A2.2.4. Model calibration*

The main outcome of interest is the overall number of infections in the hospital during the study period. Since patients with suspected infections nearly all have cultures performed, no missing infection cases were assumed.

The model was fitted separately under each identifiability scenario using Approximate Bayesian Computation (ABC). For the acceptance-rejection criteria we used the 95% Confidence Intervals (CIs). Five hundred samples (per parameter) were drawn from the parameters’ prior probability distributions using Latin-Hypercube Sampling (LHS). For each set of drawn samples, the six ICUs were simulated for the period of June 1, 2017, to July 1, 2018, using the generated event-queue data, and the simulations were repeated 30 times for each sample set. The number of simulation repetitions was determined based on the minimum number of data points required for the calculation of confidence intervals (CIs) based on the Central Limit Theorem [18]. The first 30 days of the simulations (June 1, 2017, to June 30, 2017) were discarded as the burn-in period. Each set of sampled parameters was accepted if the observed number of infections was within 95% CI of the mean of number of infections resulted from the simulations.

**A2.3. Uncertainty analysis**

For uncertainty analysis, the simulations were repeated while relaxing parameter distributions (see Table A2). We extended the upper or lower bounds of the prior parameter distributions to evaluate the sensitivity of the model to changing parameters beyond commonly observed values. This provides insight into the most influential parameters to be targeted with interventions. Using the results of the simulations, we calculated the partial rank correlation coefficients (PRCCs) for each model parameter.

We also investigated the effects of parameter distribution boundaries on PRCC calculations. For doing so, we split the uniform distribution of each parameter *p* in half, i.e., $U_{1}\left( p \right)=\left[ a, {(a+b)}/2 \right]$ and $U_{2}\left( p \right)=\left[ {(a+b)}/2, b \right]$, in which *a* and *b* are the lower and upper bounds of the parameter’s prior distribution, respectively, and repeated the PRCC analysis separately on simulations with samples drawn from $U_{1}$ and $U_{2}$. The PRCCs obtained from $U_{1}$ and $U_{2}$ (referred to as $r_{1}$ and $r_{2}$) for each model parameter were compared and tested for statistical significance using the *cocor* package [19] — an R implementation of a broad range of tests for statistical comparison of correlations.

**References**

1. Epstein JM. Agent-Based Computational Models And Generative Social Science. Complexity **1999**; 4:41–60.

2. Pedregosa, F. and Varoquaux, G. and Gramfort, A. and Michel V, and Thirion, B. and Grisel, O. and Blondel, M. and Prettenhofer P, and Weiss, R. and Dubourg, V. and Vanderplas, J. and Passos A and, Cournapeau, D. and Brucher, M. and Perrot, M. and Duchesnay E. Scikit-learn: Machine Learning in Python. J Mach Learn Res **2011**; 12:2825--2830.

3. Ziakas PD, Anagnostou T, Mylonakis E. The prevalence and significance of methicillin-resistant staphylococcus aureus colonization at admission in the general ICU setting: A meta-analysis of published studies. Crit Care Med **2014**; 42:433–444. Available at: https://journals.lww.com/ccmjournal/fulltext/2014/02000/the_prevalence_and_significance_of.24.aspx. Accessed 19 December 2023.

4. Ziakas PD, Thapa R, Rice LB, Mylonakis E. Trends and Significance of VRE Colonization in the ICU: A Meta-Analysis of Published Studies. PLoS One **2013**; 8:e75658. Available at: https://journals.plos.org/plosone/article?id=10.1371/journal.pone.0075658. Accessed 19 December 2023.

5. Luteijn JM, Hubben GAA, Pechlivanoglou P, Bonten MJ, Postma MJ. Diagnostic accuracy of culture-based and PCR-based detection tests for methicillin-resistant Staphylococcus aureus: a meta-analysis. Clin Microbiol Infect **2011**; 17:146–154.

6. Brasg I, Elligsen M, MacFadden D, Daneman N. Predictive utility of swab screening for vancomycin-resistant Enterococcus in selection of empiric antibiotics for Enterococcus sterile-site infections: a retrospective cohort study. Can Med Assoc Open Access J **2017**; 5:E632–E637. Available at: https://www.cmajopen.ca/content/5/3/E632. Accessed 19 December 2023.

7. Harris AD, Pineles L, Belton B, et al. Universal Glove and Gown Use and Acquisition of Antibiotic-Resistant Bacteria in the ICU: A Randomized Trial. JAMA **2013**; 310:1571–1580. Available at: https://jamanetwork.com/journals/jama/fullarticle/1752753. Accessed 30 June 2022.

8. Bischoff WE, Reynolds TM, Sessler CN, Edmond MB, Wenzel RP. Handwashing Compliance by Health Care Workers: The Impact of Introducing an Accessible, Alcohol-Based Hand Antiseptic. Arch Intern Med **2000**; 160:1017–1021. Available at: https://jamanetwork.com/journals/jamainternalmedicine/fullarticle/485276. Accessed 30 June 2022.

9. Rupp ME, Fitzgerald T, Puumala S, et al. Prospective, Controlled, Cross-Over Trial of Alcohol-Based Hand Gel in Critical Care Units. Infect Control Hosp Epidemiol **2008**; 29:8–15. Available at: https://www.cambridge.org/core/journals/infection-control-and-hospital-epidemiology/article/abs/prospective-controlled-crossover-trial-of-alcoholbased-hand-gel-in-critical-care-units/27AA9C54F01B1D9DD1DB89D0CA620693. Accessed 30 June 2022.

10. Reich JA, Goodstein ME, Callahan SE, et al. Physician report cards and rankings yield long-lasting hand hygiene compliance exceeding 90%. Crit Care **2015**; 19:1–6. Available at: https://ccforum.biomedcentral.com/articles/10.1186/s13054-015-1008-4. Accessed 30 June 2022.

11. Manian FA, Ponzillo JJ. Compliance With Routine Use of Gowns by Healthcare Workers (HCWs) and Non-HCW Visitors on Entry Into the Rooms of Patients Under Contact Precautions. Infect Control Hosp Epidemiol **2007**; 28:337–340. Available at: https://www.cambridge.org/core/journals/infection-control-and-hospital-epidemiology/article/abs/compliance-with-routine-use-of-gowns-by-healthcare-workers-hcws-and-nonhcw-visitors-on-entry-into-the-rooms-of-patients-under-contact-precautions/2FA9D613B74AB8E9778B999432301AF1. Accessed 30 June 2022.

12. Alhmidi H, Cadnum JL, Koganti S, et al. Shedding of methicillin-resistant Staphylococcus aureus by colonized patients during procedures and patient care activities. Infect Control Hosp Epidemiol **2019**; 40:328–332. Available at: https://www.cambridge.org/core/journals/infection-control-and-hospital-epidemiology/article/abs/shedding-of-methicillinresistant-staphylococcus-aureus-by-colonized-patients-during-procedures-and-patient-care-activities/D2713D8F846C68127BA2572FC7B6FD15. Accessed 19 December 2023.

13. Barnes SL, Myers M, Rock C, et al. Evaluating a Prediction-Driven Targeting Strategy for Reducing the Transmission of Multidrug-Resistant Organisms. https://doi.org/101287/ijoc20190916 **2020**; 32:912–929. Available at: https://pubsonline.informs.org/doi/abs/10.1287/ijoc.2019.0916. Accessed 28 June 2022.

14. Cetinkaya Y, Falk P, Mayhall CG. Vancomycin-Resistant Enterococci. Clin Microbiol Rev **2000**; 13:686–707. Available at: https://journals.asm.org/doi/10.1128/CMR.13.4.686. Accessed 21 February 2023.

15. Neely AN, Maley MP. Survival of Enterococci and Staphylococci on Hospital Fabrics and Plastic. J Clin Microbiol **2000**; 38:724–726. Available at: https://journals.asm.org/doi/10.1128/JCM.38.2.724-726.2000. Accessed 21 February 2023.

16. Wieland FG, Hauber AL, Rosenblatt M, Tönsing C, Timmer J. On structural and practical identifiability. Curr Opin Syst Biol **2021**; 25:60–69.

17. Robin TT, Cascante-Vega J, Shaman J, Pei S. System identifiability in a time-evolving agent-based model. PLoS One **2024**; 19:e0290821. Available at: https://journals.plos.org/plosone/article?id=10.1371/journal.pone.0290821. Accessed 11 March 2024.

18. Chang H-J, Huang K-C, Chao ROCROC, Wu H. Determination of Sample Size in Using Central Limit Theorem for Weibull Distribution. Inf Manag Sci **2006**; 17:31–46.

19. Diedenhofen B, Musch J. cocor: A Comprehensive Solution for the Statistical Comparison of Correlations. PLoS One **2015**; 10. Available at: /pmc/articles/PMC4383486/. Accessed 11 August 2023.
