# Supplementary material for: Using electronic medical records in hospital simulation for infection control intervention assessment
Source: Infect Control Hosp Epidemiol. 2025 Jan 9;46(3):298–304. doi: 10.1017/ice.2024.224 (PMC11883657; doi:10.1017/ice.2024.224)
Supplement: Haghpanah et al. supplementary material 2 — Haghpanah et al. supplementary material [file S0899823X24002241sup002.docx]

**Supplementary Materials for:**

Using electronic medical records in hospital simulation for infection control intervention assessment

Fardad Haghpanah^1*^ and Eili Klein^1,2^; for the CDC MInD-Healthcare Program

^1^ One Health Trust, Washington, D.C., USA

^2^ Department of Emergency Medicine, Johns Hopkins School of Medicine, Baltimore, MD, USA

^*^ Corresponding author:

5636 Connecticut Avenue NW,
PO Box 42735, Washington, D.C.
20015, U.S.A.

[haghpanah@onehealthtrust.org](mailto:haghpanah@onehealthtrust.org)

**Appendix B: Results**

**B3.1. Summary statistics of EMRs**

The infection rates were also different across the ICUs (Table B2). The MICU had both the greatest number of cases (521) and the highest infection rate (67.8 infections per 1000 patient-days). The NCCU had the lowest rate (17.2 infections per 1000 patients-days), while the CVSU had the least number of infections (81). When comparing infections by HCW visits, the MICU had the highest rate with 1.43 infections per 1,000 HCW visits and the CVSU had the lowest rate with 0.72 infections per 1,000 HCW visits.

Table B1. Capacity, patient flow, and contact rate statistics of ICUs

| **ICU** | **Capacity**  ***[beds]*** | **Daily admissions**  ***[mean (SD)]*** | **LOS (hrs.)**  ***[median (SD)]*** | **Hourly contacts**  ***[mean (SD)]*** |
| --- | --- | --- | --- | --- |
| CCU | 12 | 3.4 (1.7) | 43 (105) | 1.5 (0.8) |
| CVSU | 18 | 4.0 (2.2) | 39 (146) | 1.3 (0.5) |
| MICU | 24 | 4.8 (1.9) | 60 (163) | 2.0 (0.8) |
| NCCU | 24 | 8.1 (3.5) | 28 (89) | 0.8 (0.3) |
| SICU | 15 | 3.5 (1.8) | 26 (83) | 2.2 (1.0) |
| WSICU | 20 | 6.3 (3.1) | 31 (84) | 1.6 (0.6) |


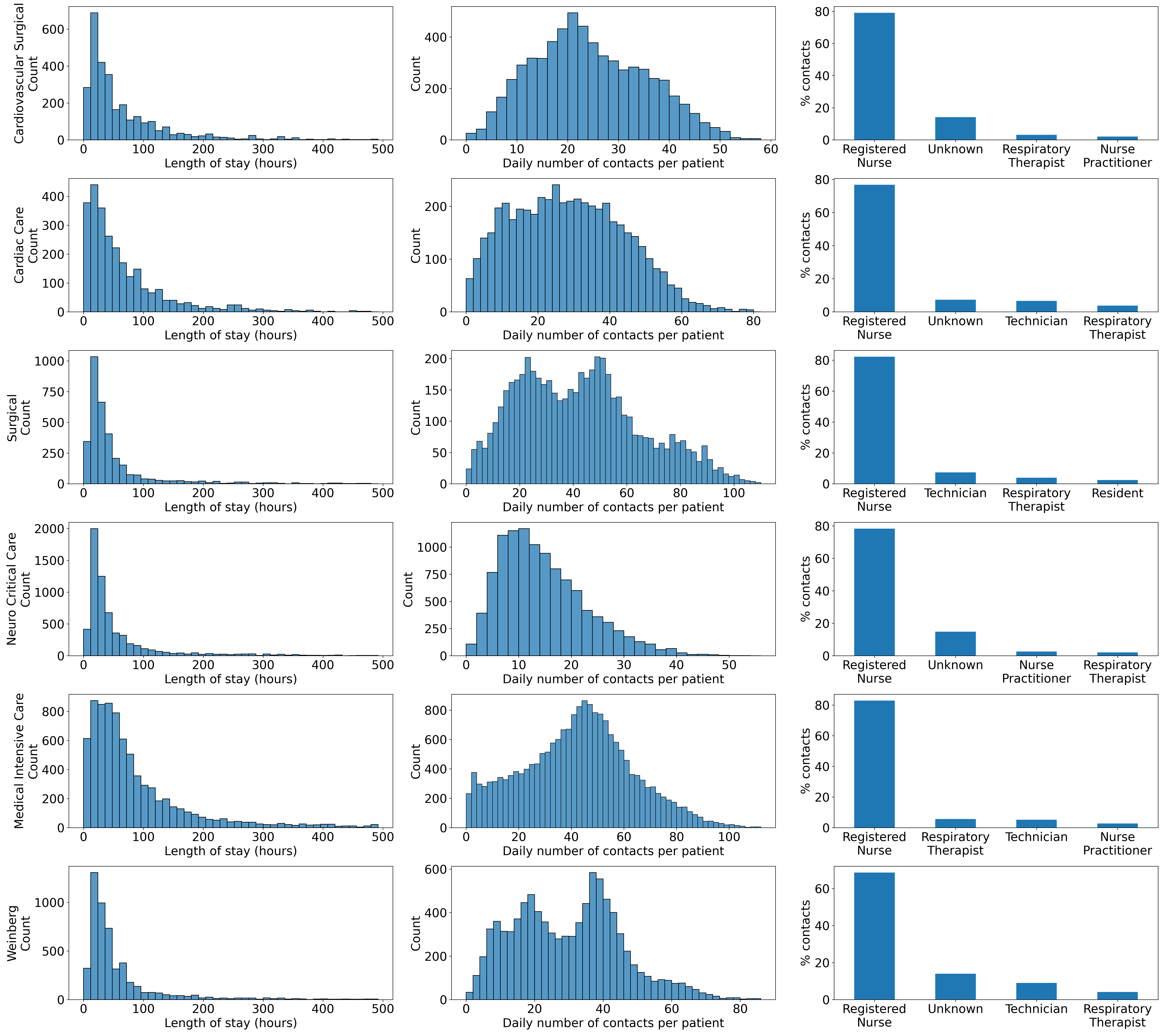


Figure B1. Distribution of length of stay, daily number of HCW contacts per patient, and the type of HCWs with the most frequent contacts in each ICU.

Table B2. MRSA/VRE infection rates in the ICUs from July 1, 2017, to July 1, 2018

| **ICU** | **Total number of infections** | **Infection rate** | **Infections per 1000 patient-days** | **Infections per 1000 HCW contacts** |
| --- | --- | --- | --- | --- |
| CCU | 139 | 11.5% | 39.56 | 1.27 |
| CVSU | 81 | 6.0% | 19.62 | 0.72 |
| MICU | 521 | 30.1% | 67.85 | 1.43 |
| NCCU | 123 | 4.2% | 17.23 | 1.06 |
| SICU | 142 | 11.6% | 54.28 | 1.18 |
| WSICU | 187 | 8.4% | 34.72 | 1.07 |
| Total | 1193 | 11.2% | 39.16 | 1.20 |

**B3.2. Calibration results**


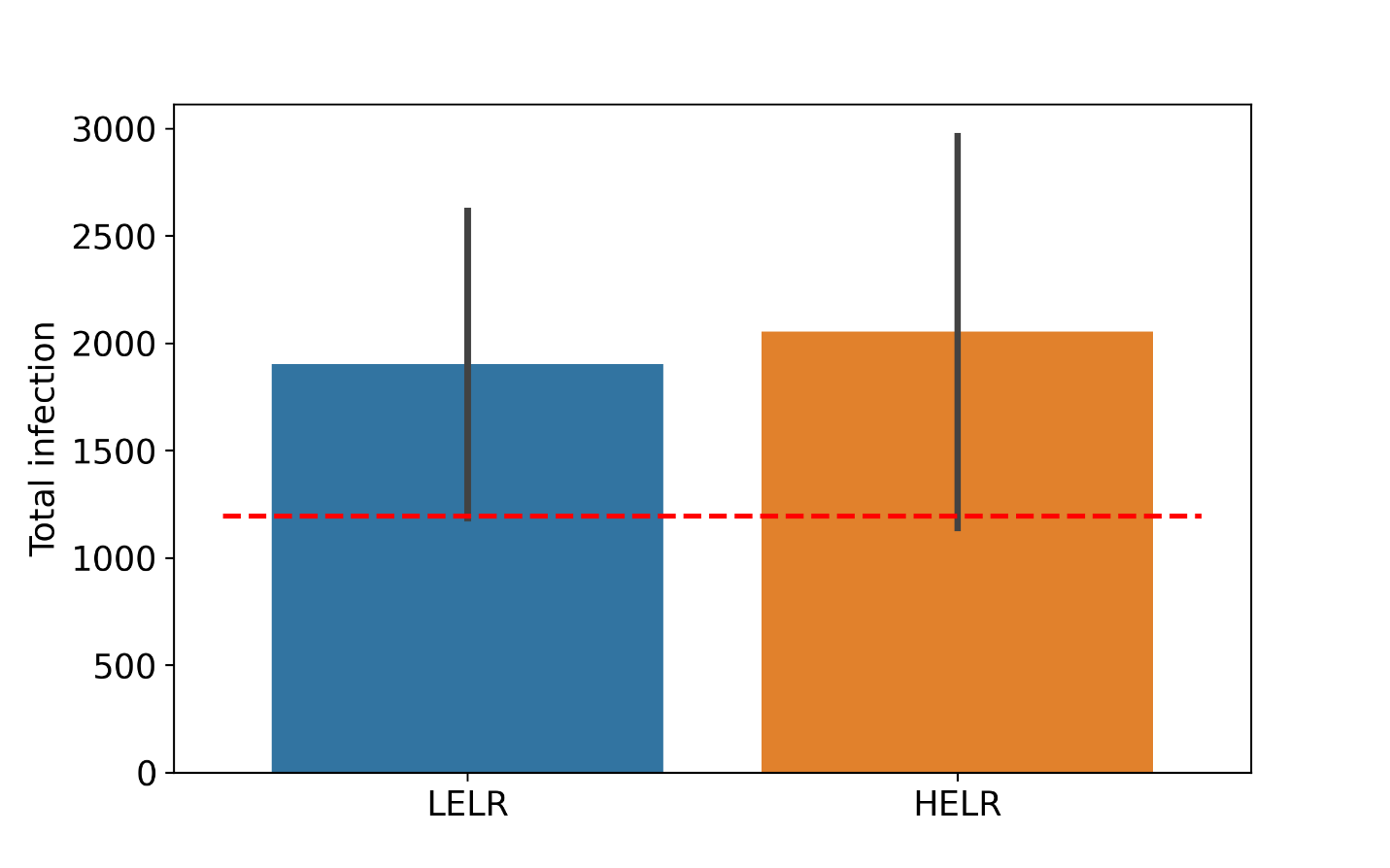


Figure B2. Simulated total number of infections resulted from the posterior samples for each scenario. The horizontal dashed line shows the observed number of infection cases used in the calibration process. The bars point to the average number of infections from the accepted samples, with the 95% confidence intervals shown with the solid vertical lines on bars.

**B3.3. Results of uncertainty analysis**

A common approach in Systems Biology for global uncertainty analysis of complex models is correlation-based sensitivity analysis using partial rank correlation coefficients (PRCCs) [1]. PRCC is a sampling-based method and is suitable to analyze monotonic relationships (linear or nonlinear) between a model’s input and output parameters, after discounting for the effects of randomness in the remaining input parameters.


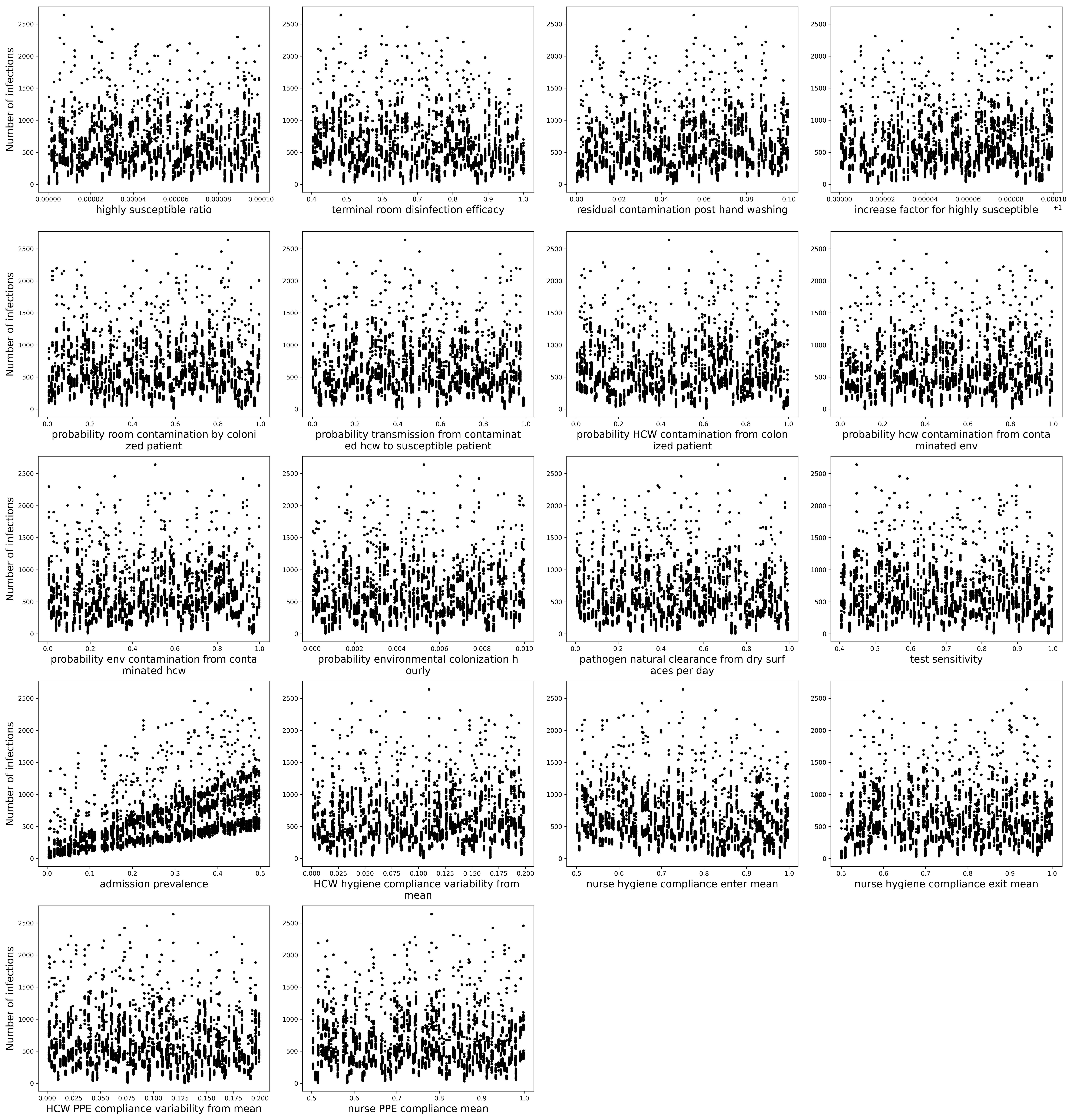


Figure B3. Scatter plots of model parameters versus model output (i.e., number of infections) over all the ICUs.

Table B3. Partial rank correlation coefficients for all model parameters in each ICU under the LELR scenario (in descending order of strength)

| **Parameter** | **PRCC** | **ICU** |
| --- | --- | --- |
| admission prevalence | 0.971*** | CCU |
| residual contamination post hand washing | 0.468*** | CCU |
| nurse hygiene compliance enter mean | -0.457*** | CCU |
| nurse hygiene compliance exit mean | -0.419*** | CCU |
| terminal room disinfection efficacy | -0.282** | CCU |
| probability env contamination from contaminated hcw | 0.245** | CCU |
| probability hcw contamination from contaminated env | 0.225** | CCU |
| probability HCW contamination from colonized patient | 0.221** | CCU |
| pathogen natural clearance from dry surfaces per day | 0.128 | CCU |
| HCW PPE compliance variability from mean | -0.118 | CCU |
| test sensitivity | -0.102 | CCU |
| HCW hygiene compliance variability from mean | 0.098 | CCU |
| probability environmental colonization hourly | 0.05 | CCU |
| nurse PPE compliance mean | 0.027 | CCU |
| probability transmission from contaminated hcw to susceptible patient | -0.023 | CCU |
| highly susceptible ratio | 0.018 | CCU |
| increase factor for highly susceptible | -0.008 | CCU |
| probability room contamination by colonized patient | -0.005 | CCU |
| admission prevalence | 0.984*** | CVSU |
| residual contamination post hand washing | 0.505*** | CVSU |
| nurse hygiene compliance enter mean | -0.351*** | CVSU |
| terminal room disinfection efficacy | -0.288** | CVSU |
| nurse hygiene compliance exit mean | -0.259** | CVSU |
| probability HCW contamination from colonized patient | 0.177 | CVSU |
| probability hcw contamination from contaminated env | 0.167 | CVSU |
| HCW PPE compliance variability from mean | -0.16 | CVSU |
| probability env contamination from contaminated hcw | 0.145 | CVSU |
| pathogen natural clearance from dry surfaces per day | 0.13 | CVSU |
| HCW hygiene compliance variability from mean | 0.106 | CVSU |
| test sensitivity | -0.081 | CVSU |
| probability transmission from contaminated hcw to susceptible patient | -0.066 | CVSU |
| nurse PPE compliance mean | 0.065 | CVSU |
| probability room contamination by colonized patient | 0.058 | CVSU |
| increase factor for highly susceptible | -0.053 | CVSU |
| highly susceptible ratio | 0.019 | CVSU |
| probability environmental colonization hourly | 0.017 | CVSU |
| admission prevalence | 0.929*** | MICU |
| residual contamination post hand washing | 0.558*** | MICU |
| nurse hygiene compliance enter mean | -0.518*** | MICU |
| nurse hygiene compliance exit mean | -0.483*** | MICU |
| probability env contamination from contaminated hcw | 0.347*** | MICU |
| terminal room disinfection efficacy | -0.277** | MICU |
| probability hcw contamination from contaminated env | 0.277** | MICU |
| probability HCW contamination from colonized patient | 0.171 | MICU |
| HCW PPE compliance variability from mean | -0.145 | MICU |
| pathogen natural clearance from dry surfaces per day | 0.137 | MICU |
| test sensitivity | -0.13 | MICU |
| probability transmission from contaminated hcw to susceptible patient | -0.089 | MICU |
| HCW hygiene compliance variability from mean | 0.088 | MICU |
| increase factor for highly susceptible | -0.083 | MICU |
| probability environmental colonization hourly | 0.07 | MICU |
| nurse PPE compliance mean | -0.04 | MICU |
| highly susceptible ratio | 0.023 | MICU |
| probability room contamination by colonized patient | 0.001 | MICU |
| admission prevalence | 0.987*** | NCCU |
| residual contamination post hand washing | 0.356*** | NCCU |
| nurse hygiene compliance enter mean | -0.315*** | NCCU |
| terminal room disinfection efficacy | -0.304*** | NCCU |
| nurse hygiene compliance exit mean | -0.228** | NCCU |
| probability HCW contamination from colonized patient | 0.176 | NCCU |
| probability hcw contamination from contaminated env | 0.167 | NCCU |
| pathogen natural clearance from dry surfaces per day | 0.146 | NCCU |
| HCW PPE compliance variability from mean | -0.145 | NCCU |
| probability env contamination from contaminated hcw | 0.1 | NCCU |
| nurse PPE compliance mean | 0.082 | NCCU |
| probability room contamination by colonized patient | 0.072 | NCCU |
| test sensitivity | -0.069 | NCCU |
| HCW hygiene compliance variability from mean | 0.055 | NCCU |
| highly susceptible ratio | 0.049 | NCCU |
| probability transmission from contaminated hcw to susceptible patient | -0.037 | NCCU |
| probability environmental colonization hourly | 0.032 | NCCU |
| increase factor for highly susceptible | -0.025 | NCCU |
| admission prevalence | 0.977*** | SICU |
| residual contamination post hand washing | 0.52*** | SICU |
| nurse hygiene compliance enter mean | -0.434*** | SICU |
| nurse hygiene compliance exit mean | -0.339*** | SICU |
| terminal room disinfection efficacy | -0.302*** | SICU |
| probability HCW contamination from colonized patient | 0.218* | SICU |
| probability env contamination from contaminated hcw | 0.193* | SICU |
| pathogen natural clearance from dry surfaces per day | 0.17 | SICU |
| HCW PPE compliance variability from mean | -0.165 | SICU |
| probability hcw contamination from contaminated env | 0.143 | SICU |
| test sensitivity | -0.121 | SICU |
| HCW hygiene compliance variability from mean | 0.078 | SICU |
| probability environmental colonization hourly | 0.062 | SICU |
| probability transmission from contaminated hcw to susceptible patient | -0.058 | SICU |
| nurse PPE compliance mean | 0.049 | SICU |
| probability room contamination by colonized patient | 0.03 | SICU |
| increase factor for highly susceptible | -0.026 | SICU |
| highly susceptible ratio | 0.01 | SICU |
| admission prevalence | 0.975*** | WSICU |
| residual contamination post hand washing | 0.465*** | WSICU |
| nurse hygiene compliance enter mean | -0.435*** | WSICU |
| nurse hygiene compliance exit mean | -0.38*** | WSICU |
| terminal room disinfection efficacy | -0.314*** | WSICU |
| probability env contamination from contaminated hcw | 0.233** | WSICU |
| probability hcw contamination from contaminated env | 0.22* | WSICU |
| probability HCW contamination from colonized patient | 0.195* | WSICU |
| pathogen natural clearance from dry surfaces per day | 0.139 | WSICU |
| test sensitivity | -0.134 | WSICU |
| HCW PPE compliance variability from mean | -0.129 | WSICU |
| HCW hygiene compliance variability from mean | 0.086 | WSICU |
| probability environmental colonization hourly | 0.053 | WSICU |
| nurse PPE compliance mean | 0.053 | WSICU |
| probability room contamination by colonized patient | 0.031 | WSICU |
| highly susceptible ratio | 0.028 | WSICU |
| probability transmission from contaminated hcw to susceptible patient | -0.024 | WSICU |
| increase factor for highly susceptible | 0 | WSICU |

(***) Significant at the 1% level; (**) Significant at the 5% level; (*) Significant at the 10% level.

Table B4. Partial rank correlation coefficients for all model parameters in each ICU under the LEHR scenario (in descending order of strength)

| **Parameter** | **PRCC** | **ICU** |
| --- | --- | --- |
| admission prevalence | 0.944*** | CCU |
| terminal room disinfection efficacy | -0.296*** | CCU |
| residual contamination post hand washing | 0.225** | CCU |
| probability HCW contamination from colonized patient | 0.187* | CCU |
| probability hcw contamination from contaminated env | 0.174 | CCU |
| pathogen natural clearance from dry surfaces per day | 0.151 | CCU |
| HCW PPE compliance variability from mean | -0.1 | CCU |
| highly susceptible ratio | 0.098 | CCU |
| probability room contamination by colonized patient | -0.081 | CCU |
| test sensitivity | -0.072 | CCU |
| nurse PPE compliance mean | 0.059 | CCU |
| HCW hygiene compliance variability from mean | 0.05 | CCU |
| nurse hygiene compliance exit mean | -0.029 | CCU |
| increase factor for highly susceptible | -0.023 | CCU |
| probability transmission from contaminated hcw to susceptible patient | -0.022 | CCU |
| nurse hygiene compliance enter mean | -0.021 | CCU |
| probability environmental colonization hourly | 0.01 | CCU |
| probability env contamination from contaminated hcw | -0.007 | CCU |
| admission prevalence | 0.956*** | CVSU |
| terminal room disinfection efficacy | -0.298*** | CVSU |
| residual contamination post hand washing | 0.264** | CVSU |
| pathogen natural clearance from dry surfaces per day | 0.163 | CVSU |
| probability HCW contamination from colonized patient | 0.159 | CVSU |
| probability hcw contamination from contaminated env | 0.125 | CVSU |
| highly susceptible ratio | 0.099 | CVSU |
| probability room contamination by colonized patient | -0.088 | CVSU |
| probability env contamination from contaminated hcw | -0.085 | CVSU |
| HCW PPE compliance variability from mean | -0.068 | CVSU |
| nurse PPE compliance mean | 0.055 | CVSU |
| nurse hygiene compliance enter mean | -0.041 | CVSU |
| test sensitivity | -0.035 | CVSU |
| probability environmental colonization hourly | 0.029 | CVSU |
| HCW hygiene compliance variability from mean | 0.029 | CVSU |
| probability transmission from contaminated hcw to susceptible patient | -0.023 | CVSU |
| increase factor for highly susceptible | -0.013 | CVSU |
| nurse hygiene compliance exit mean | 0.009 | CVSU |
| admission prevalence | 0.853*** | MICU |
| terminal room disinfection efficacy | -0.297*** | MICU |
| residual contamination post hand washing | 0.188* | MICU |
| pathogen natural clearance from dry surfaces per day | 0.177 | MICU |
| probability HCW contamination from colonized patient | 0.163 | MICU |
| probability hcw contamination from contaminated env | 0.137 | MICU |
| probability room contamination by colonized patient | -0.127 | MICU |
| highly susceptible ratio | 0.089 | MICU |
| HCW hygiene compliance variability from mean | 0.077 | MICU |
| HCW PPE compliance variability from mean | -0.064 | MICU |
| nurse PPE compliance mean | 0.055 | MICU |
| test sensitivity | -0.047 | MICU |
| probability transmission from contaminated hcw to susceptible patient | -0.038 | MICU |
| increase factor for highly susceptible | -0.034 | MICU |
| probability environmental colonization hourly | 0.031 | MICU |
| probability env contamination from contaminated hcw | -0.02 | MICU |
| nurse hygiene compliance exit mean | -0.019 | MICU |
| nurse hygiene compliance enter mean | 0.007 | MICU |
| admission prevalence | 0.977*** | NCCU |
| terminal room disinfection efficacy | -0.339*** | NCCU |
| probability HCW contamination from colonized patient | 0.185 | NCCU |
| residual contamination post hand washing | 0.18 | NCCU |
| pathogen natural clearance from dry surfaces per day | 0.169 | NCCU |
| probability hcw contamination from contaminated env | 0.126 | NCCU |
| nurse hygiene compliance enter mean | -0.113 | NCCU |
| HCW PPE compliance variability from mean | -0.102 | NCCU |
| highly susceptible ratio | 0.073 | NCCU |
| test sensitivity | -0.067 | NCCU |
| nurse hygiene compliance exit mean | -0.066 | NCCU |
| HCW hygiene compliance variability from mean | 0.063 | NCCU |
| nurse PPE compliance mean | 0.044 | NCCU |
| probability environmental colonization hourly | 0.032 | NCCU |
| probability transmission from contaminated hcw to susceptible patient | -0.02 | NCCU |
| probability env contamination from contaminated hcw | -0.015 | NCCU |
| probability room contamination by colonized patient | -0.007 | NCCU |
| increase factor for highly susceptible | 0.007 | NCCU |
| admission prevalence | 0.946*** | SICU |
| terminal room disinfection efficacy | -0.341*** | SICU |
| residual contamination post hand washing | 0.262** | SICU |
| pathogen natural clearance from dry surfaces per day | 0.213* | SICU |
| probability HCW contamination from colonized patient | 0.182 | SICU |
| probability hcw contamination from contaminated env | 0.154 | SICU |
| nurse PPE compliance mean | 0.093 | SICU |
| HCW hygiene compliance variability from mean | 0.081 | SICU |
| HCW PPE compliance variability from mean | -0.078 | SICU |
| probability room contamination by colonized patient | -0.075 | SICU |
| test sensitivity | -0.056 | SICU |
| highly susceptible ratio | 0.053 | SICU |
| nurse hygiene compliance exit mean | -0.052 | SICU |
| probability environmental colonization hourly | 0.048 | SICU |
| probability env contamination from contaminated hcw | -0.043 | SICU |
| nurse hygiene compliance enter mean | -0.033 | SICU |
| probability transmission from contaminated hcw to susceptible patient | -0.018 | SICU |
| increase factor for highly susceptible | -0.015 | SICU |
| admission prevalence | 0.948*** | WSICU |
| terminal room disinfection efficacy | -0.358*** | WSICU |
| residual contamination post hand washing | 0.235** | WSICU |
| probability HCW contamination from colonized patient | 0.202* | WSICU |
| pathogen natural clearance from dry surfaces per day | 0.17 | WSICU |
| probability hcw contamination from contaminated env | 0.146 | WSICU |
| HCW PPE compliance variability from mean | -0.088 | WSICU |
| test sensitivity | -0.087 | WSICU |
| nurse hygiene compliance enter mean | -0.085 | WSICU |
| highly susceptible ratio | 0.079 | WSICU |
| nurse PPE compliance mean | 0.075 | WSICU |
| probability room contamination by colonized patient | -0.075 | WSICU |
| nurse hygiene compliance exit mean | -0.069 | WSICU |
| HCW hygiene compliance variability from mean | 0.06 | WSICU |
| probability environmental colonization hourly | 0.023 | WSICU |
| increase factor for highly susceptible | 0.01 | WSICU |
| probability transmission from contaminated hcw to susceptible patient | -0.009 | WSICU |
| probability env contamination from contaminated hcw | -0.005 | WSICU |

(***) Significant at the 1% level; (**) Significant at the 5% level; (*) Significant at the 10% level.

Table B5. Partial rank correlation coefficients for all model parameters in each ICU under the HELR scenario (in descending order of strength)

| **Parameter** | **PRCC** | **ICU** |
| --- | --- | --- |
| admission prevalence | 0.961*** | CCU |
| nurse hygiene compliance enter mean | -0.641*** | CCU |
| nurse hygiene compliance exit mean | -0.557*** | CCU |
| residual contamination post hand washing | 0.394*** | CCU |
| probability env contamination from contaminated hcw | 0.393*** | CCU |
| terminal room disinfection efficacy | -0.242** | CCU |
| test sensitivity | -0.237** | CCU |
| HCW PPE compliance variability from mean | -0.195* | CCU |
| probability HCW contamination from colonized patient | 0.184 | CCU |
| probability hcw contamination from contaminated env | 0.13 | CCU |
| pathogen natural clearance from dry surfaces per day | 0.111 | CCU |
| increase factor for highly susceptible | -0.092 | CCU |
| HCW hygiene compliance variability from mean | 0.069 | CCU |
| nurse PPE compliance mean | -0.044 | CCU |
| probability transmission from contaminated hcw to susceptible patient | -0.04 | CCU |
| highly susceptible ratio | -0.036 | CCU |
| probability environmental colonization hourly | 0.026 | CCU |
| probability room contamination by colonized patient | 0.016 | CCU |
| admission prevalence | 0.98*** | CVSU |
| nurse hygiene compliance enter mean | -0.587*** | CVSU |
| nurse hygiene compliance exit mean | -0.475*** | CVSU |
| residual contamination post hand washing | 0.449*** | CVSU |
| probability env contamination from contaminated hcw | 0.292*** | CVSU |
| HCW PPE compliance variability from mean | -0.24** | CVSU |
| terminal room disinfection efficacy | -0.234** | CVSU |
| test sensitivity | -0.216* | CVSU |
| probability HCW contamination from colonized patient | 0.156 | CVSU |
| pathogen natural clearance from dry surfaces per day | 0.113 | CVSU |
| probability room contamination by colonized patient | 0.094 | CVSU |
| highly susceptible ratio | -0.06 | CVSU |
| HCW hygiene compliance variability from mean | 0.06 | CVSU |
| increase factor for highly susceptible | -0.059 | CVSU |
| probability hcw contamination from contaminated env | 0.038 | CVSU |
| probability transmission from contaminated hcw to susceptible patient | -0.03 | CVSU |
| nurse PPE compliance mean | 0.029 | CVSU |
| probability environmental colonization hourly | 0.006 | CVSU |
| admission prevalence | 0.911*** | MICU |
| nurse hygiene compliance enter mean | -0.66*** | MICU |
| nurse hygiene compliance exit mean | -0.608*** | MICU |
| probability env contamination from contaminated hcw | 0.495*** | MICU |
| residual contamination post hand washing | 0.489*** | MICU |
| terminal room disinfection efficacy | -0.272** | MICU |
| test sensitivity | -0.218* | MICU |
| HCW PPE compliance variability from mean | -0.212* | MICU |
| probability HCW contamination from colonized patient | 0.152 | MICU |
| pathogen natural clearance from dry surfaces per day | 0.148 | MICU |
| increase factor for highly susceptible | -0.145 | MICU |
| probability hcw contamination from contaminated env | 0.106 | MICU |
| nurse PPE compliance mean | -0.087 | MICU |
| probability transmission from contaminated hcw to susceptible patient | -0.06 | MICU |
| highly susceptible ratio | -0.018 | MICU |
| probability room contamination by colonized patient | 0.015 | MICU |
| HCW hygiene compliance variability from mean | 0.01 | MICU |
| probability environmental colonization hourly | -0.001 | MICU |
| admission prevalence | 0.984*** | NCCU |
| nurse hygiene compliance enter mean | -0.55*** | NCCU |
| nurse hygiene compliance exit mean | -0.436*** | NCCU |
| terminal room disinfection efficacy | -0.279** | NCCU |
| probability env contamination from contaminated hcw | 0.264** | NCCU |
| test sensitivity | -0.254** | NCCU |
| HCW PPE compliance variability from mean | -0.222** | NCCU |
| residual contamination post hand washing | 0.218* | NCCU |
| probability HCW contamination from colonized patient | 0.139 | NCCU |
| pathogen natural clearance from dry surfaces per day | 0.109 | NCCU |
| probability room contamination by colonized patient | 0.1 | NCCU |
| HCW hygiene compliance variability from mean | 0.095 | NCCU |
| probability hcw contamination from contaminated env | 0.058 | NCCU |
| highly susceptible ratio | -0.049 | NCCU |
| increase factor for highly susceptible | -0.041 | NCCU |
| probability transmission from contaminated hcw to susceptible patient | 0.019 | NCCU |
| nurse PPE compliance mean | 0.012 | NCCU |
| probability environmental colonization hourly | -0.003 | NCCU |
| admission prevalence | 0.972*** | SICU |
| nurse hygiene compliance enter mean | -0.594*** | SICU |
| nurse hygiene compliance exit mean | -0.506*** | SICU |
| residual contamination post hand washing | 0.482*** | SICU |
| probability env contamination from contaminated hcw | 0.337*** | SICU |
| terminal room disinfection efficacy | -0.281** | SICU |
| test sensitivity | -0.271** | SICU |
| HCW PPE compliance variability from mean | -0.224** | SICU |
| probability HCW contamination from colonized patient | 0.169 | SICU |
| pathogen natural clearance from dry surfaces per day | 0.147 | SICU |
| probability hcw contamination from contaminated env | 0.107 | SICU |
| increase factor for highly susceptible | -0.09 | SICU |
| HCW hygiene compliance variability from mean | 0.062 | SICU |
| probability room contamination by colonized patient | 0.061 | SICU |
| highly susceptible ratio | -0.056 | SICU |
| probability transmission from contaminated hcw to susceptible patient | -0.032 | SICU |
| nurse PPE compliance mean | 0.022 | SICU |
| probability environmental colonization hourly | -0.001 | SICU |
| admission prevalence | 0.966*** | WSICU |
| nurse hygiene compliance enter mean | -0.618*** | WSICU |
| nurse hygiene compliance exit mean | -0.545*** | WSICU |
| probability env contamination from contaminated hcw | 0.37*** | WSICU |
| residual contamination post hand washing | 0.362*** | WSICU |
| terminal room disinfection efficacy | -0.312*** | WSICU |
| test sensitivity | -0.25** | WSICU |
| HCW PPE compliance variability from mean | -0.217* | WSICU |
| probability HCW contamination from colonized patient | 0.173 | WSICU |
| pathogen natural clearance from dry surfaces per day | 0.139 | WSICU |
| probability hcw contamination from contaminated env | 0.099 | WSICU |
| HCW hygiene compliance variability from mean | 0.064 | WSICU |
| probability room contamination by colonized patient | 0.062 | WSICU |
| increase factor for highly susceptible | -0.051 | WSICU |
| highly susceptible ratio | -0.024 | WSICU |
| nurse PPE compliance mean | 0.017 | WSICU |
| probability transmission from contaminated hcw to susceptible patient | 0.01 | WSICU |
| probability environmental colonization hourly | -0.001 | WSICU |

(***) Significant at the 1% level; (**) Significant at the 5% level; (*) Significant at the 10% level.

Table B6. Partial rank correlation coefficients for all model parameters in each ICU under the HEHR scenario (in descending order of strength)

| **Parameter** | **PRCC** | **ICU** |
| --- | --- | --- |
| admission prevalence | 0.923*** | CCU |
| terminal room disinfection efficacy | -0.376*** | CCU |
| nurse hygiene compliance enter mean | -0.282** | CCU |
| nurse hygiene compliance exit mean | -0.251** | CCU |
| probability HCW contamination from colonized patient | 0.238** | CCU |
| residual contamination post hand washing | 0.191* | CCU |
| probability env contamination from contaminated hcw | 0.183 | CCU |
| pathogen natural clearance from dry surfaces per day | 0.163 | CCU |
| HCW PPE compliance variability from mean | -0.126 | CCU |
| probability hcw contamination from contaminated env | 0.12 | CCU |
| highly susceptible ratio | 0.075 | CCU |
| probability environmental colonization hourly | -0.07 | CCU |
| test sensitivity | -0.061 | CCU |
| increase factor for highly susceptible | -0.056 | CCU |
| probability room contamination by colonized patient | -0.054 | CCU |
| HCW hygiene compliance variability from mean | 0.049 | CCU |
| nurse PPE compliance mean | 0.036 | CCU |
| probability transmission from contaminated hcw to susceptible patient | -0.013 | CCU |
| admission prevalence | 0.942*** | CVSU |
| terminal room disinfection efficacy | -0.325*** | CVSU |
| pathogen natural clearance from dry surfaces per day | 0.197* | CVSU |
| residual contamination post hand washing | 0.197* | CVSU |
| nurse hygiene compliance enter mean | -0.195* | CVSU |
| probability HCW contamination from colonized patient | 0.188* | CVSU |
| nurse hygiene compliance exit mean | -0.175 | CVSU |
| HCW PPE compliance variability from mean | -0.118 | CVSU |
| probability hcw contamination from contaminated env | 0.108 | CVSU |
| nurse PPE compliance mean | 0.099 | CVSU |
| probability environmental colonization hourly | -0.094 | CVSU |
| probability env contamination from contaminated hcw | 0.088 | CVSU |
| highly susceptible ratio | 0.073 | CVSU |
| probability room contamination by colonized patient | -0.066 | CVSU |
| test sensitivity | -0.063 | CVSU |
| increase factor for highly susceptible | -0.049 | CVSU |
| HCW hygiene compliance variability from mean | 0.019 | CVSU |
| probability transmission from contaminated hcw to susceptible patient | -0.005 | CVSU |
| admission prevalence | 0.821*** | MICU |
| terminal room disinfection efficacy | -0.358*** | MICU |
| nurse hygiene compliance exit mean | -0.193* | MICU |
| residual contamination post hand washing | 0.175 | MICU |
| probability HCW contamination from colonized patient | 0.165 | MICU |
| pathogen natural clearance from dry surfaces per day | 0.165 | MICU |
| nurse hygiene compliance enter mean | -0.16 | MICU |
| probability env contamination from contaminated hcw | 0.157 | MICU |
| probability hcw contamination from contaminated env | 0.146 | MICU |
| highly susceptible ratio | 0.102 | MICU |
| probability environmental colonization hourly | -0.099 | MICU |
| nurse PPE compliance mean | 0.086 | MICU |
| HCW PPE compliance variability from mean | -0.084 | MICU |
| increase factor for highly susceptible | -0.082 | MICU |
| probability room contamination by colonized patient | -0.072 | MICU |
| test sensitivity | -0.061 | MICU |
| HCW hygiene compliance variability from mean | 0.028 | MICU |
| probability transmission from contaminated hcw to susceptible patient | -0.022 | MICU |
| admission prevalence | 0.97*** | NCCU |
| terminal room disinfection efficacy | -0.418*** | NCCU |
| nurse hygiene compliance enter mean | -0.291*** | NCCU |
| nurse hygiene compliance exit mean | -0.29*** | NCCU |
| probability HCW contamination from colonized patient | 0.26** | NCCU |
| pathogen natural clearance from dry surfaces per day | 0.213* | NCCU |
| residual contamination post hand washing | 0.162 | NCCU |
| probability env contamination from contaminated hcw | 0.158 | NCCU |
| HCW PPE compliance variability from mean | -0.148 | NCCU |
| test sensitivity | -0.127 | NCCU |
| probability environmental colonization hourly | -0.123 | NCCU |
| probability hcw contamination from contaminated env | 0.109 | NCCU |
| highly susceptible ratio | 0.074 | NCCU |
| HCW hygiene compliance variability from mean | 0.058 | NCCU |
| nurse PPE compliance mean | 0.048 | NCCU |
| increase factor for highly susceptible | -0.03 | NCCU |
| probability transmission from contaminated hcw to susceptible patient | 0.02 | NCCU |
| probability room contamination by colonized patient | -0.011 | NCCU |
| admission prevalence | 0.922*** | SICU |
| terminal room disinfection efficacy | -0.417*** | SICU |
| nurse hygiene compliance exit mean | -0.222** | SICU |
| probability HCW contamination from colonized patient | 0.215* | SICU |
| residual contamination post hand washing | 0.207* | SICU |
| nurse hygiene compliance enter mean | -0.201* | SICU |
| pathogen natural clearance from dry surfaces per day | 0.167 | SICU |
| probability env contamination from contaminated hcw | 0.146 | SICU |
| HCW PPE compliance variability from mean | -0.125 | SICU |
| probability hcw contamination from contaminated env | 0.119 | SICU |
| test sensitivity | -0.104 | SICU |
| nurse PPE compliance mean | 0.097 | SICU |
| probability environmental colonization hourly | -0.095 | SICU |
| highly susceptible ratio | 0.079 | SICU |
| HCW hygiene compliance variability from mean | 0.054 | SICU |
| probability room contamination by colonized patient | -0.037 | SICU |
| increase factor for highly susceptible | -0.019 | SICU |
| probability transmission from contaminated hcw to susceptible patient | -0.007 | SICU |
| admission prevalence | 0.929*** | WSICU |
| terminal room disinfection efficacy | -0.453*** | WSICU |
| nurse hygiene compliance exit mean | -0.305*** | WSICU |
| nurse hygiene compliance enter mean | -0.275** | WSICU |
| probability HCW contamination from colonized patient | 0.222** | WSICU |
| residual contamination post hand washing | 0.203* | WSICU |
| pathogen natural clearance from dry surfaces per day | 0.19* | WSICU |
| probability env contamination from contaminated hcw | 0.171 | WSICU |
| HCW PPE compliance variability from mean | -0.139 | WSICU |
| test sensitivity | -0.125 | WSICU |
| probability hcw contamination from contaminated env | 0.101 | WSICU |
| nurse PPE compliance mean | 0.091 | WSICU |
| probability environmental colonization hourly | -0.085 | WSICU |
| HCW hygiene compliance variability from mean | 0.073 | WSICU |
| highly susceptible ratio | 0.073 | WSICU |
| probability room contamination by colonized patient | -0.05 | WSICU |
| increase factor for highly susceptible | -0.041 | WSICU |
| probability transmission from contaminated hcw to susceptible patient | -0.008 | WSICU |

(***) Significant at the 1% level; (**) Significant at the 5% level; (*) Significant at the 10% level.

Table B7. Sensitivity of distribution boundaries on partial rank correlation coefficients for all model parameters in each ICU under the LELR scenario (in descending order of significance)

|  | | | | **P values** | | | | |
| --- | --- | --- | --- | --- | --- | --- | --- | --- |
| **Parameter** | **PRCC_1_** | **PRCC_2_** | **ICU** | **Pearson** | **Dunn** | **Steiger** | **Raghunathan** | **Silver** |
| increase factor for highly susceptible | 0.212 | -0.228 | CCU | <0.01 | <0.01 | <0.01 | <0.01 | <0.01 |
| probability env contamination from contaminated hcw | 0.453 | 0.156 | CCU | <0.01 | <0.01 | <0.01 | <0.01 | <0.01 |
| HCW PPE compliance variability from mean | -0.163 | 0.096 | CCU | <0.01 | <0.01 | <0.01 | <0.01 | <0.01 |
| probability transmission from contaminated hcw to susceptible patient | -0.165 | 0.087 | CCU | <0.01 | <0.01 | <0.01 | <0.01 | <0.01 |
| nurse hygiene compliance exit mean | -0.202 | -0.426 | CCU | <0.01 | <0.01 | <0.01 | <0.01 | <0.01 |
| probability room contamination by colonized patient | -0.036 | -0.246 | CCU | <0.01 | <0.01 | <0.01 | <0.01 | <0.01 |
| probability hcw contamination from contaminated env | 0.181 | 0.38 | CCU | <0.01 | <0.01 | <0.01 | <0.01 | <0.01 |
| HCW hygiene compliance variability from mean | 0.217 | 0.403 | CCU | <0.01 | <0.01 | <0.01 | <0.01 | <0.01 |
| pathogen natural clearance from dry surfaces per day | 0.01 | 0.193 | CCU | <0.01 | <0.01 | <0.01 | <0.01 | <0.01 |
| probability environmental colonization hourly | 0.205 | 0.023 | CCU | <0.01 | <0.01 | <0.01 | <0.01 | <0.01 |
| nurse hygiene compliance enter mean | -0.237 | -0.414 | CCU | <0.01 | <0.01 | <0.01 | <0.01 | <0.01 |
| terminal room disinfection efficacy | -0.19 | -0.324 | CCU | <0.01 | <0.01 | <0.01 | <0.01 | <0.01 |
| highly susceptible ratio | 0.088 | 0.191 | CCU | <0.01 | <0.01 | <0.01 | <0.01 | <0.01 |
| nurse PPE compliance mean | 0.231 | 0.333 | CCU | <0.01 | <0.01 | <0.01 | <0.01 | <0.01 |
| probability HCW contamination from colonized patient | 0.023 | 0.079 | CCU | 0.078 | 0.079 | 0.079 | 0.079 | 0.079 |
| admission prevalence | 0.919 | 0.871 | CCU | <0.01 | <0.01 | <0.01 | <0.01 | <0.01 |
| residual contamination post hand washing | 0.143 | 0.105 | CCU | >0.1 | >0.1 | >0.1 | >0.1 | >0.1 |
| test sensitivity | -0.01 | 0.025 | CCU | >0.1 | >0.1 | >0.1 | >0.1 | >0.1 |
| probability hcw contamination from contaminated env | 0.02 | 0.463 | CVSU | <0.01 | <0.01 | <0.01 | <0.01 | <0.01 |
| probability transmission from contaminated hcw to susceptible patient | -0.253 | 0.174 | CVSU | <0.01 | <0.01 | <0.01 | <0.01 | <0.01 |
| increase factor for highly susceptible | 0.114 | -0.223 | CVSU | <0.01 | <0.01 | <0.01 | <0.01 | <0.01 |
| HCW hygiene compliance variability from mean | 0.099 | 0.429 | CVSU | <0.01 | <0.01 | <0.01 | <0.01 | <0.01 |
| nurse hygiene compliance exit mean | -0.052 | -0.326 | CVSU | <0.01 | <0.01 | <0.01 | <0.01 | <0.01 |
| terminal room disinfection efficacy | -0.112 | -0.375 | CVSU | <0.01 | <0.01 | <0.01 | <0.01 | <0.01 |
| nurse hygiene compliance enter mean | -0.194 | -0.408 | CVSU | <0.01 | <0.01 | <0.01 | <0.01 | <0.01 |
| highly susceptible ratio | -0.017 | 0.17 | CVSU | <0.01 | <0.01 | <0.01 | <0.01 | <0.01 |
| probability room contamination by colonized patient | 0.034 | -0.142 | CVSU | <0.01 | <0.01 | <0.01 | <0.01 | <0.01 |
| test sensitivity | -0.077 | 0.075 | CVSU | <0.01 | <0.01 | <0.01 | <0.01 | <0.01 |
| probability env contamination from contaminated hcw | 0.256 | 0.126 | CVSU | <0.01 | <0.01 | <0.01 | <0.01 | <0.01 |
| probability environmental colonization hourly | 0.069 | -0.061 | CVSU | <0.01 | <0.01 | <0.01 | <0.01 | <0.01 |
| HCW PPE compliance variability from mean | -0.119 | -0.003 | CVSU | <0.01 | <0.01 | <0.01 | <0.01 | <0.01 |
| pathogen natural clearance from dry surfaces per day | 0.093 | 0.192 | CVSU | <0.01 | <0.01 | <0.01 | <0.01 | <0.01 |
| nurse PPE compliance mean | 0.32 | 0.248 | CVSU | 0.014 | 0.014 | 0.014 | 0.014 | 0.014 |
| probability HCW contamination from colonized patient | -0.009 | 0.062 | CVSU | 0.024 | 0.024 | 0.024 | 0.024 | 0.024 |
| admission prevalence | 0.953 | 0.922 | CVSU | <0.01 | <0.01 | <0.01 | <0.01 | <0.01 |
| residual contamination post hand washing | 0.13 | 0.111 | CVSU | >0.1 | >0.1 | >0.1 | >0.1 | >0.1 |
| increase factor for highly susceptible | 0.232 | -0.263 | MICU | <0.01 | <0.01 | <0.01 | <0.01 | <0.01 |
| probability env contamination from contaminated hcw | 0.584 | 0.157 | MICU | <0.01 | <0.01 | <0.01 | <0.01 | <0.01 |
| HCW PPE compliance variability from mean | -0.229 | 0.101 | MICU | <0.01 | <0.01 | <0.01 | <0.01 | <0.01 |
| nurse hygiene compliance enter mean | -0.197 | -0.499 | MICU | <0.01 | <0.01 | <0.01 | <0.01 | <0.01 |
| nurse hygiene compliance exit mean | -0.21 | -0.465 | MICU | <0.01 | <0.01 | <0.01 | <0.01 | <0.01 |
| nurse PPE compliance mean | 0.117 | 0.338 | MICU | <0.01 | <0.01 | <0.01 | <0.01 | <0.01 |
| pathogen natural clearance from dry surfaces per day | 0.007 | 0.185 | MICU | <0.01 | <0.01 | <0.01 | <0.01 | <0.01 |
| probability environmental colonization hourly | 0.215 | 0.053 | MICU | <0.01 | <0.01 | <0.01 | <0.01 | <0.01 |
| probability room contamination by colonized patient | -0.148 | -0.29 | MICU | <0.01 | <0.01 | <0.01 | <0.01 | <0.01 |
| admission prevalence | 0.855 | 0.736 | MICU | <0.01 | <0.01 | <0.01 | <0.01 | <0.01 |
| probability HCW contamination from colonized patient | -0.027 | 0.06 | MICU | <0.01 | <0.01 | <0.01 | <0.01 | <0.01 |
| highly susceptible ratio | 0.144 | 0.21 | MICU | 0.029 | 0.029 | 0.029 | 0.029 | 0.029 |
| probability hcw contamination from contaminated env | 0.347 | 0.289 | MICU | 0.042 | 0.042 | 0.042 | 0.042 | 0.042 |
| probability transmission from contaminated hcw to susceptible patient | -0.086 | -0.047 | MICU | >0.1 | >0.1 | >0.1 | >0.1 | >0.1 |
| terminal room disinfection efficacy | -0.246 | -0.282 | MICU | >0.1 | >0.1 | >0.1 | >0.1 | >0.1 |
| residual contamination post hand washing | 0.239 | 0.251 | MICU | >0.1 | >0.1 | >0.1 | >0.1 | >0.1 |
| HCW hygiene compliance variability from mean | 0.34 | 0.348 | MICU | >0.1 | >0.1 | >0.1 | >0.1 | >0.1 |
| test sensitivity | -0.007 | -0.005 | MICU | >0.1 | >0.1 | >0.1 | >0.1 | >0.1 |
| probability hcw contamination from contaminated env | -0.006 | 0.513 | NCCU | <0.01 | <0.01 | <0.01 | <0.01 | <0.01 |
| terminal room disinfection efficacy | 0.013 | -0.413 | NCCU | <0.01 | <0.01 | <0.01 | <0.01 | <0.01 |
| probability transmission from contaminated hcw to susceptible patient | -0.244 | 0.113 | NCCU | <0.01 | <0.01 | <0.01 | <0.01 | <0.01 |
| HCW hygiene compliance variability from mean | 0.058 | 0.393 | NCCU | <0.01 | <0.01 | <0.01 | <0.01 | <0.01 |
| nurse hygiene compliance exit mean | -0.061 | -0.281 | NCCU | <0.01 | <0.01 | <0.01 | <0.01 | <0.01 |
| nurse hygiene compliance enter mean | -0.206 | -0.411 | NCCU | <0.01 | <0.01 | <0.01 | <0.01 | <0.01 |
| probability environmental colonization hourly | 0.126 | -0.034 | NCCU | <0.01 | <0.01 | <0.01 | <0.01 | <0.01 |
| probability env contamination from contaminated hcw | 0.175 | 0.077 | NCCU | <0.01 | <0.01 | <0.01 | <0.01 | <0.01 |
| highly susceptible ratio | -0.08 | -0.17 | NCCU | <0.01 | <0.01 | <0.01 | <0.01 | <0.01 |
| test sensitivity | -0.097 | -0.009 | NCCU | <0.01 | <0.01 | <0.01 | <0.01 | <0.01 |
| probability room contamination by colonized patient | 0.011 | -0.068 | NCCU | 0.013 | 0.014 | 0.014 | 0.014 | 0.014 |
| probability HCW contamination from colonized patient | -0.073 | 0.001 | NCCU | 0.019 | 0.019 | 0.019 | 0.019 | 0.019 |
| nurse PPE compliance mean | 0.341 | 0.274 | NCCU | 0.019 | 0.019 | 0.019 | 0.019 | 0.019 |
| HCW PPE compliance variability from mean | -0.052 | 0.012 | NCCU | 0.046 | 0.046 | 0.046 | 0.046 | 0.046 |
| increase factor for highly susceptible | 0.061 | 0.12 | NCCU | 0.057 | 0.057 | 0.057 | 0.057 | 0.057 |
| residual contamination post hand washing | 0.049 | 0.02 | NCCU | >0.1 | >0.1 | >0.1 | >0.1 | >0.1 |
| admission prevalence | 0.961 | 0.944 | NCCU | <0.01 | <0.01 | <0.01 | <0.01 | <0.01 |
| pathogen natural clearance from dry surfaces per day | 0.183 | 0.178 | NCCU | >0.1 | >0.1 | >0.1 | >0.1 | >0.1 |
| increase factor for highly susceptible | 0.151 | -0.195 | SICU | <0.01 | <0.01 | <0.01 | <0.01 | <0.01 |
| probability hcw contamination from contaminated env | 0.066 | 0.41 | SICU | <0.01 | <0.01 | <0.01 | <0.01 | <0.01 |
| nurse hygiene compliance exit mean | -0.107 | -0.359 | SICU | <0.01 | <0.01 | <0.01 | <0.01 | <0.01 |
| probability env contamination from contaminated hcw | 0.345 | 0.098 | SICU | <0.01 | <0.01 | <0.01 | <0.01 | <0.01 |
| probability environmental colonization hourly | 0.199 | -0.036 | SICU | <0.01 | <0.01 | <0.01 | <0.01 | <0.01 |
| probability transmission from contaminated hcw to susceptible patient | -0.152 | 0.066 | SICU | <0.01 | <0.01 | <0.01 | <0.01 | <0.01 |
| HCW PPE compliance variability from mean | -0.139 | 0.07 | SICU | <0.01 | <0.01 | <0.01 | <0.01 | <0.01 |
| terminal room disinfection efficacy | -0.161 | -0.367 | SICU | <0.01 | <0.01 | <0.01 | <0.01 | <0.01 |
| HCW hygiene compliance variability from mean | 0.209 | 0.393 | SICU | <0.01 | <0.01 | <0.01 | <0.01 | <0.01 |
| nurse hygiene compliance enter mean | -0.27 | -0.432 | SICU | <0.01 | <0.01 | <0.01 | <0.01 | <0.01 |
| probability room contamination by colonized patient | -0.046 | -0.186 | SICU | <0.01 | <0.01 | <0.01 | <0.01 | <0.01 |
| probability HCW contamination from colonized patient | -0.07 | 0.051 | SICU | <0.01 | <0.01 | <0.01 | <0.01 | <0.01 |
| pathogen natural clearance from dry surfaces per day | 0.095 | 0.189 | SICU | <0.01 | <0.01 | <0.01 | <0.01 | <0.01 |
| test sensitivity | -0.058 | -0.002 | SICU | 0.076 | 0.076 | 0.076 | 0.076 | 0.076 |
| highly susceptible ratio | 0.076 | 0.123 | SICU | >0.1 | >0.1 | >0.1 | >0.1 | >0.1 |
| nurse PPE compliance mean | 0.257 | 0.296 | SICU | >0.1 | >0.1 | >0.1 | >0.1 | >0.1 |
| admission prevalence | 0.934 | 0.897 | SICU | <0.01 | <0.01 | <0.01 | <0.01 | <0.01 |
| residual contamination post hand washing | 0.176 | 0.161 | SICU | >0.1 | >0.1 | >0.1 | >0.1 | >0.1 |
| increase factor for highly susceptible | 0.148 | -0.184 | WSICU | <0.01 | <0.01 | <0.01 | <0.01 | <0.01 |
| probability hcw contamination from contaminated env | 0.166 | 0.44 | WSICU | <0.01 | <0.01 | <0.01 | <0.01 | <0.01 |
| HCW PPE compliance variability from mean | -0.135 | 0.131 | WSICU | <0.01 | <0.01 | <0.01 | <0.01 | <0.01 |
| probability environmental colonization hourly | 0.222 | -0.042 | WSICU | <0.01 | <0.01 | <0.01 | <0.01 | <0.01 |
| probability env contamination from contaminated hcw | 0.393 | 0.132 | WSICU | <0.01 | <0.01 | <0.01 | <0.01 | <0.01 |
| probability transmission from contaminated hcw to susceptible patient | -0.126 | 0.09 | WSICU | <0.01 | <0.01 | <0.01 | <0.01 | <0.01 |
| probability room contamination by colonized patient | 0.011 | -0.198 | WSICU | <0.01 | <0.01 | <0.01 | <0.01 | <0.01 |
| nurse hygiene compliance enter mean | -0.263 | -0.467 | WSICU | <0.01 | <0.01 | <0.01 | <0.01 | <0.01 |
| highly susceptible ratio | 0.056 | -0.146 | WSICU | <0.01 | <0.01 | <0.01 | <0.01 | <0.01 |
| HCW hygiene compliance variability from mean | 0.22 | 0.403 | WSICU | <0.01 | <0.01 | <0.01 | <0.01 | <0.01 |
| nurse hygiene compliance exit mean | -0.176 | -0.346 | WSICU | <0.01 | <0.01 | <0.01 | <0.01 | <0.01 |
| nurse PPE compliance mean | 0.198 | 0.335 | WSICU | <0.01 | <0.01 | <0.01 | <0.01 | <0.01 |
| terminal room disinfection efficacy | -0.218 | -0.348 | WSICU | <0.01 | <0.01 | <0.01 | <0.01 | <0.01 |
| residual contamination post hand washing | 0.187 | 0.08 | WSICU | <0.01 | <0.01 | <0.01 | <0.01 | <0.01 |
| probability HCW contamination from colonized patient | -0.038 | 0.035 | WSICU | 0.022 | 0.023 | 0.023 | 0.023 | 0.023 |
| pathogen natural clearance from dry surfaces per day | 0.116 | 0.185 | WSICU | 0.025 | 0.025 | 0.025 | 0.025 | 0.025 |
| test sensitivity | -0.053 | 0.013 | WSICU | 0.039 | 0.039 | 0.039 | 0.039 | 0.039 |
| admission prevalence | 0.927 | 0.889 | WSICU | <0.01 | <0.01 | <0.01 | <0.01 | <0.01 |

Table B8. Sensitivity of distribution boundaries on partial rank correlation coefficients for all model parameters in each ICU under the LEHR scenario (in descending order of significance)

|  | | | | **P values** | | | | |
| --- | --- | --- | --- | --- | --- | --- | --- | --- |
| **Parameter** | **PRCC_1_** | **PRCC_2_** | **ICU** | **Pearson** | **Dunn** | **Steiger** | **Raghunathan** | **Silver** |
| terminal room disinfection efficacy | -0.072 | -0.432 | CCU | <0.01 | <0.01 | <0.01 | <0.01 | <0.01 |
| increase factor for highly susceptible | 0.083 | -0.219 | CCU | <0.01 | <0.01 | <0.01 | <0.01 | <0.01 |
| nurse hygiene compliance enter mean | -0.009 | -0.275 | CCU | <0.01 | <0.01 | <0.01 | <0.01 | <0.01 |
| probability hcw contamination from contaminated env | 0.157 | 0.419 | CCU | <0.01 | <0.01 | <0.01 | <0.01 | <0.01 |
| nurse hygiene compliance exit mean | 0.086 | -0.155 | CCU | <0.01 | <0.01 | <0.01 | <0.01 | <0.01 |
| probability transmission from contaminated hcw to susceptible patient | -0.134 | 0.009 | CCU | <0.01 | <0.01 | <0.01 | <0.01 | <0.01 |
| probability env contamination from contaminated hcw | 0.094 | -0.008 | CCU | <0.01 | <0.01 | <0.01 | <0.01 | <0.01 |
| probability HCW contamination from colonized patient | -0.049 | 0.045 | CCU | <0.01 | <0.01 | <0.01 | <0.01 | <0.01 |
| HCW hygiene compliance variability from mean | 0.206 | 0.292 | CCU | <0.01 | <0.01 | <0.01 | <0.01 | <0.01 |
| HCW PPE compliance variability from mean | 0.006 | 0.088 | CCU | <0.01 | <0.01 | <0.01 | <0.01 | <0.01 |
| nurse PPE compliance mean | 0.254 | 0.176 | CCU | 0.01 | 0.01 | 0.01 | 0.01 | 0.01 |
| test sensitivity | -0.063 | 0.01 | CCU | 0.02 | 0.02 | 0.02 | 0.02 | 0.02 |
| probability room contamination by colonized patient | -0.104 | -0.176 | CCU | 0.02 | 0.02 | 0.02 | 0.02 | 0.02 |
| highly susceptible ratio | 0.157 | 0.088 | CCU | 0.027 | 0.027 | 0.027 | 0.027 | 0.027 |
| pathogen natural clearance from dry surfaces per day | 0.118 | 0.072 | CCU | >0.1 | >0.1 | >0.1 | >0.1 | >0.1 |
| residual contamination post hand washing | -0.12 | -0.079 | CCU | >0.1 | >0.1 | >0.1 | >0.1 | >0.1 |
| probability environmental colonization hourly | 0.108 | 0.074 | CCU | >0.1 | >0.1 | >0.1 | >0.1 | >0.1 |
| admission prevalence | 0.852 | 0.843 | CCU | >0.1 | >0.1 | >0.1 | >0.1 | >0.1 |
| probability hcw contamination from contaminated env | 0.075 | 0.403 | CVSU | <0.01 | <0.01 | <0.01 | <0.01 | <0.01 |
| increase factor for highly susceptible | 0.088 | -0.236 | CVSU | <0.01 | <0.01 | <0.01 | <0.01 | <0.01 |
| terminal room disinfection efficacy | -0.089 | -0.411 | CVSU | <0.01 | <0.01 | <0.01 | <0.01 | <0.01 |
| nurse hygiene compliance enter mean | 0.018 | -0.246 | CVSU | <0.01 | <0.01 | <0.01 | <0.01 | <0.01 |
| probability transmission from contaminated hcw to susceptible patient | -0.199 | 0.023 | CVSU | <0.01 | <0.01 | <0.01 | <0.01 | <0.01 |
| nurse hygiene compliance exit mean | 0.142 | -0.072 | CVSU | <0.01 | <0.01 | <0.01 | <0.01 | <0.01 |
| pathogen natural clearance from dry surfaces per day | 0.198 | 0.021 | CVSU | <0.01 | <0.01 | <0.01 | <0.01 | <0.01 |
| nurse PPE compliance mean | 0.257 | 0.128 | CVSU | <0.01 | <0.01 | <0.01 | <0.01 | <0.01 |
| test sensitivity | -0.038 | 0.088 | CVSU | <0.01 | <0.01 | <0.01 | <0.01 | <0.01 |
| HCW hygiene compliance variability from mean | 0.181 | 0.283 | CVSU | <0.01 | <0.01 | <0.01 | <0.01 | <0.01 |
| probability HCW contamination from colonized patient | -0.043 | 0.04 | CVSU | <0.01 | <0.01 | <0.01 | <0.01 | <0.01 |
| probability env contamination from contaminated hcw | -0.002 | -0.064 | CVSU | 0.049 | 0.05 | 0.05 | 0.05 | 0.05 |
| highly susceptible ratio | 0.164 | 0.117 | CVSU | >0.1 | >0.1 | >0.1 | >0.1 | >0.1 |
| probability room contamination by colonized patient | -0.096 | -0.142 | CVSU | >0.1 | >0.1 | >0.1 | >0.1 | >0.1 |
| probability environmental colonization hourly | 0.046 | 0.011 | CVSU | >0.1 | >0.1 | >0.1 | >0.1 | >0.1 |
| residual contamination post hand washing | -0.099 | -0.084 | CVSU | >0.1 | >0.1 | >0.1 | >0.1 | >0.1 |
| admission prevalence | 0.874 | 0.886 | CVSU | 0.094 | 0.093 | 0.093 | 0.093 | 0.093 |
| HCW PPE compliance variability from mean | 0.116 | 0.115 | CVSU | >0.1 | >0.1 | >0.1 | >0.1 | >0.1 |
| terminal room disinfection efficacy | -0.092 | -0.443 | MICU | <0.01 | <0.01 | <0.01 | <0.01 | <0.01 |
| nurse hygiene compliance enter mean | 0.004 | -0.266 | MICU | <0.01 | <0.01 | <0.01 | <0.01 | <0.01 |
| nurse hygiene compliance exit mean | 0.117 | -0.12 | MICU | <0.01 | <0.01 | <0.01 | <0.01 | <0.01 |
| increase factor for highly susceptible | 0.033 | -0.198 | MICU | <0.01 | <0.01 | <0.01 | <0.01 | <0.01 |
| probability hcw contamination from contaminated env | 0.163 | 0.373 | MICU | <0.01 | <0.01 | <0.01 | <0.01 | <0.01 |
| probability transmission from contaminated hcw to susceptible patient | -0.208 | -0.017 | MICU | <0.01 | <0.01 | <0.01 | <0.01 | <0.01 |
| pathogen natural clearance from dry surfaces per day | 0.201 | 0.066 | MICU | <0.01 | <0.01 | <0.01 | <0.01 | <0.01 |
| highly susceptible ratio | 0.195 | 0.077 | MICU | <0.01 | <0.01 | <0.01 | <0.01 | <0.01 |
| nurse PPE compliance mean | 0.208 | 0.095 | MICU | <0.01 | <0.01 | <0.01 | <0.01 | <0.01 |
| probability HCW contamination from colonized patient | -0.066 | 0.024 | MICU | <0.01 | <0.01 | <0.01 | <0.01 | <0.01 |
| HCW hygiene compliance variability from mean | 0.193 | 0.281 | MICU | <0.01 | <0.01 | <0.01 | <0.01 | <0.01 |
| test sensitivity | -0.075 | 0.001 | MICU | 0.017 | 0.017 | 0.017 | 0.017 | 0.017 |
| probability room contamination by colonized patient | -0.111 | -0.18 | MICU | 0.025 | 0.025 | 0.025 | 0.025 | 0.025 |
| probability environmental colonization hourly | 0.193 | 0.125 | MICU | 0.026 | 0.026 | 0.026 | 0.026 | 0.026 |
| HCW PPE compliance variability from mean | 0.118 | 0.073 | MICU | >0.1 | >0.1 | >0.1 | >0.1 | >0.1 |
| probability env contamination from contaminated hcw | 0.03 | 0.01 | MICU | >0.1 | >0.1 | >0.1 | >0.1 | >0.1 |
| residual contamination post hand washing | -0.136 | -0.154 | MICU | >0.1 | >0.1 | >0.1 | >0.1 | >0.1 |
| admission prevalence | 0.698 | 0.687 | MICU | >0.1 | >0.1 | >0.1 | >0.1 | >0.1 |
| probability hcw contamination from contaminated env | 0.069 | 0.455 | NCCU | <0.01 | <0.01 | <0.01 | <0.01 | <0.01 |
| terminal room disinfection efficacy | -0.099 | -0.436 | NCCU | <0.01 | <0.01 | <0.01 | <0.01 | <0.01 |
| probability transmission from contaminated hcw to susceptible patient | -0.261 | 0.061 | NCCU | <0.01 | <0.01 | <0.01 | <0.01 | <0.01 |
| increase factor for highly susceptible | 0.041 | -0.195 | NCCU | <0.01 | <0.01 | <0.01 | <0.01 | <0.01 |
| HCW hygiene compliance variability from mean | 0.129 | 0.355 | NCCU | <0.01 | <0.01 | <0.01 | <0.01 | <0.01 |
| nurse hygiene compliance enter mean | -0.083 | -0.302 | NCCU | <0.01 | <0.01 | <0.01 | <0.01 | <0.01 |
| nurse hygiene compliance exit mean | 0.034 | -0.183 | NCCU | <0.01 | <0.01 | <0.01 | <0.01 | <0.01 |
| probability room contamination by colonized patient | -0.03 | -0.143 | NCCU | <0.01 | <0.01 | <0.01 | <0.01 | <0.01 |
| nurse PPE compliance mean | 0.302 | 0.194 | NCCU | <0.01 | <0.01 | <0.01 | <0.01 | <0.01 |
| test sensitivity | -0.093 | -0.007 | NCCU | <0.01 | <0.01 | <0.01 | <0.01 | <0.01 |
| probability HCW contamination from colonized patient | -0.035 | 0.047 | NCCU | <0.01 | <0.01 | <0.01 | <0.01 | <0.01 |
| HCW PPE compliance variability from mean | 0.005 | 0.068 | NCCU | 0.047 | 0.047 | 0.047 | 0.047 | 0.047 |
| probability environmental colonization hourly | 0.088 | 0.049 | NCCU | >0.1 | >0.1 | >0.1 | >0.1 | >0.1 |
| pathogen natural clearance from dry surfaces per day | 0.166 | 0.129 | NCCU | >0.1 | >0.1 | >0.1 | >0.1 | >0.1 |
| residual contamination post hand washing | -0.089 | -0.124 | NCCU | >0.1 | >0.1 | >0.1 | >0.1 | >0.1 |
| probability env contamination from contaminated hcw | 0.026 | -0.008 | NCCU | >0.1 | >0.1 | >0.1 | >0.1 | >0.1 |
| highly susceptible ratio | 0.11 | 0.087 | NCCU | >0.1 | >0.1 | >0.1 | >0.1 | >0.1 |
| admission prevalence | 0.928 | 0.925 | NCCU | >0.1 | >0.1 | >0.1 | >0.1 | >0.1 |
| probability hcw contamination from contaminated env | 0.161 | 0.472 | SICU | <0.01 | <0.01 | <0.01 | <0.01 | <0.01 |
| nurse hygiene compliance enter mean | -0.007 | -0.308 | SICU | <0.01 | <0.01 | <0.01 | <0.01 | <0.01 |
| terminal room disinfection efficacy | -0.12 | -0.418 | SICU | <0.01 | <0.01 | <0.01 | <0.01 | <0.01 |
| increase factor for highly susceptible | 0.05 | -0.232 | SICU | <0.01 | <0.01 | <0.01 | <0.01 | <0.01 |
| probability transmission from contaminated hcw to susceptible patient | -0.201 | 0.024 | SICU | <0.01 | <0.01 | <0.01 | <0.01 | <0.01 |
| HCW hygiene compliance variability from mean | 0.135 | 0.346 | SICU | <0.01 | <0.01 | <0.01 | <0.01 | <0.01 |
| nurse hygiene compliance exit mean | 0.073 | -0.125 | SICU | <0.01 | <0.01 | <0.01 | <0.01 | <0.01 |
| test sensitivity | -0.103 | 0.065 | SICU | <0.01 | <0.01 | <0.01 | <0.01 | <0.01 |
| pathogen natural clearance from dry surfaces per day | 0.224 | 0.064 | SICU | <0.01 | <0.01 | <0.01 | <0.01 | <0.01 |
| probability room contamination by colonized patient | -0.053 | -0.148 | SICU | <0.01 | <0.01 | <0.01 | <0.01 | <0.01 |
| probability HCW contamination from colonized patient | -0.08 | -0.02 | SICU | 0.058 | 0.059 | 0.059 | 0.059 | 0.059 |
| probability environmental colonization hourly | 0.113 | 0.064 | SICU | >0.1 | >0.1 | >0.1 | >0.1 | >0.1 |
| probability env contamination from contaminated hcw | 0.038 | -0.006 | SICU | >0.1 | >0.1 | >0.1 | >0.1 | >0.1 |
| residual contamination post hand washing | -0.111 | -0.069 | SICU | >0.1 | >0.1 | >0.1 | >0.1 | >0.1 |
| highly susceptible ratio | 0.121 | 0.086 | SICU | >0.1 | >0.1 | >0.1 | >0.1 | >0.1 |
| admission prevalence | 0.854 | 0.826 | SICU | <0.01 | <0.01 | <0.01 | <0.01 | <0.01 |
| HCW PPE compliance variability from mean | 0.086 | 0.064 | SICU | >0.1 | >0.1 | >0.1 | >0.1 | >0.1 |
| nurse PPE compliance mean | 0.234 | 0.233 | SICU | >0.1 | >0.1 | >0.1 | >0.1 | >0.1 |
| terminal room disinfection efficacy | -0.122 | -0.435 | WSICU | <0.01 | <0.01 | <0.01 | <0.01 | <0.01 |
| increase factor for highly susceptible | 0.093 | -0.22 | WSICU | <0.01 | <0.01 | <0.01 | <0.01 | <0.01 |
| probability hcw contamination from contaminated env | 0.154 | 0.416 | WSICU | <0.01 | <0.01 | <0.01 | <0.01 | <0.01 |
| nurse hygiene compliance exit mean | 0.106 | -0.155 | WSICU | <0.01 | <0.01 | <0.01 | <0.01 | <0.01 |
| nurse hygiene compliance enter mean | -0.038 | -0.263 | WSICU | <0.01 | <0.01 | <0.01 | <0.01 | <0.01 |
| probability transmission from contaminated hcw to susceptible patient | -0.145 | 0.02 | WSICU | <0.01 | <0.01 | <0.01 | <0.01 | <0.01 |
| probability room contamination by colonized patient | -0.049 | -0.197 | WSICU | <0.01 | <0.01 | <0.01 | <0.01 | <0.01 |
| probability environmental colonization hourly | 0.163 | 0.021 | WSICU | <0.01 | <0.01 | <0.01 | <0.01 | <0.01 |
| HCW hygiene compliance variability from mean | 0.175 | 0.282 | WSICU | <0.01 | <0.01 | <0.01 | <0.01 | <0.01 |
| test sensitivity | -0.068 | 0.027 | WSICU | <0.01 | <0.01 | <0.01 | <0.01 | <0.01 |
| highly susceptible ratio | 0.171 | 0.089 | WSICU | <0.01 | <0.01 | <0.01 | <0.01 | <0.01 |
| probability HCW contamination from colonized patient | -0.041 | 0.036 | WSICU | 0.015 | 0.015 | 0.015 | 0.015 | 0.015 |
| pathogen natural clearance from dry surfaces per day | 0.185 | 0.111 | WSICU | 0.017 | 0.018 | 0.018 | 0.018 | 0.018 |
| probability env contamination from contaminated hcw | 0.056 | 0.006 | WSICU | >0.1 | >0.1 | >0.1 | >0.1 | >0.1 |
| HCW PPE compliance variability from mean | 0.049 | 0.095 | WSICU | >0.1 | >0.1 | >0.1 | >0.1 | >0.1 |
| nurse PPE compliance mean | 0.256 | 0.224 | WSICU | >0.1 | >0.1 | >0.1 | >0.1 | >0.1 |
| residual contamination post hand washing | -0.096 | -0.114 | WSICU | >0.1 | >0.1 | >0.1 | >0.1 | >0.1 |
| admission prevalence | 0.857 | 0.846 | WSICU | >0.1 | >0.1 | >0.1 | >0.1 | >0.1 |

Table B9. Sensitivity of distribution boundaries on partial rank correlation coefficients for all model parameters in each ICU under the HELR scenario (in descending order of significance)

|  | | | | **P values** | | | | |
| --- | --- | --- | --- | --- | --- | --- | --- | --- |
| **Parameter** | **PRCC_1_** | **PRCC_2_** | **ICU** | **Pearson** | **Dunn** | **Steiger** | **Raghunathan** | **Silver** |
| probability env contamination from contaminated hcw | 0.626 | 0.084 | CCU | <0.01 | <0.01 | <0.01 | <0.01 | <0.01 |
| HCW PPE compliance variability from mean | -0.296 | 0.088 | CCU | <0.01 | <0.01 | <0.01 | <0.01 | <0.01 |
| increase factor for highly susceptible | 0.205 | -0.16 | CCU | <0.01 | <0.01 | <0.01 | <0.01 | <0.01 |
| pathogen natural clearance from dry surfaces per day | 0.02 | 0.35 | CCU | <0.01 | <0.01 | <0.01 | <0.01 | <0.01 |
| nurse hygiene compliance exit mean | -0.26 | -0.552 | CCU | <0.01 | <0.01 | <0.01 | <0.01 | <0.01 |
| HCW hygiene compliance variability from mean | 0.115 | 0.376 | CCU | <0.01 | <0.01 | <0.01 | <0.01 | <0.01 |
| probability environmental colonization hourly | 0.236 | 0.002 | CCU | <0.01 | <0.01 | <0.01 | <0.01 | <0.01 |
| probability room contamination by colonized patient | -0.049 | -0.251 | CCU | <0.01 | <0.01 | <0.01 | <0.01 | <0.01 |
| nurse PPE compliance mean | 0.144 | 0.345 | CCU | <0.01 | <0.01 | <0.01 | <0.01 | <0.01 |
| residual contamination post hand washing | 0.255 | 0.124 | CCU | <0.01 | <0.01 | <0.01 | <0.01 | <0.01 |
| test sensitivity | -0.167 | -0.056 | CCU | <0.01 | <0.01 | <0.01 | <0.01 | <0.01 |
| probability hcw contamination from contaminated env | 0.139 | 0.249 | CCU | <0.01 | <0.01 | <0.01 | <0.01 | <0.01 |
| probability HCW contamination from colonized patient | 0.082 | -0.013 | CCU | <0.01 | <0.01 | <0.01 | <0.01 | <0.01 |
| nurse hygiene compliance enter mean | -0.524 | -0.438 | CCU | <0.01 | <0.01 | <0.01 | <0.01 | <0.01 |
| highly susceptible ratio | 0.107 | 0.034 | CCU | 0.02 | 0.02 | 0.02 | 0.02 | 0.02 |
| probability transmission from contaminated hcw to susceptible patient | -0.052 | 0.02 | CCU | 0.023 | 0.023 | 0.023 | 0.023 | 0.023 |
| admission prevalence | 0.892 | 0.848 | CCU | <0.01 | <0.01 | <0.01 | <0.01 | <0.01 |
| terminal room disinfection efficacy | -0.176 | -0.213 | CCU | >0.1 | >0.1 | >0.1 | >0.1 | >0.1 |
| HCW hygiene compliance variability from mean | -0.05 | 0.44 | CVSU | <0.01 | <0.01 | <0.01 | <0.01 | <0.01 |
| probability env contamination from contaminated hcw | 0.498 | 0.081 | CVSU | <0.01 | <0.01 | <0.01 | <0.01 | <0.01 |
| pathogen natural clearance from dry surfaces per day | 0.01 | 0.372 | CVSU | <0.01 | <0.01 | <0.01 | <0.01 | <0.01 |
| probability hcw contamination from contaminated env | -0.055 | 0.284 | CVSU | <0.01 | <0.01 | <0.01 | <0.01 | <0.01 |
| nurse hygiene compliance exit mean | -0.198 | -0.462 | CVSU | <0.01 | <0.01 | <0.01 | <0.01 | <0.01 |
| HCW PPE compliance variability from mean | -0.289 | -0.064 | CVSU | <0.01 | <0.01 | <0.01 | <0.01 | <0.01 |
| probability room contamination by colonized patient | 0.082 | -0.141 | CVSU | <0.01 | <0.01 | <0.01 | <0.01 | <0.01 |
| residual contamination post hand washing | 0.297 | 0.098 | CVSU | <0.01 | <0.01 | <0.01 | <0.01 | <0.01 |
| increase factor for highly susceptible | 0.037 | -0.141 | CVSU | <0.01 | <0.01 | <0.01 | <0.01 | <0.01 |
| probability transmission from contaminated hcw to susceptible patient | -0.125 | 0.042 | CVSU | <0.01 | <0.01 | <0.01 | <0.01 | <0.01 |
| nurse hygiene compliance enter mean | -0.57 | -0.425 | CVSU | <0.01 | <0.01 | <0.01 | <0.01 | <0.01 |
| test sensitivity | -0.209 | -0.074 | CVSU | <0.01 | <0.01 | <0.01 | <0.01 | <0.01 |
| probability HCW contamination from colonized patient | -0.141 | -0.014 | CVSU | <0.01 | <0.01 | <0.01 | <0.01 | <0.01 |
| probability environmental colonization hourly | 0.141 | 0.019 | CVSU | <0.01 | <0.01 | <0.01 | <0.01 | <0.01 |
| nurse PPE compliance mean | 0.257 | 0.323 | CVSU | 0.022 | 0.022 | 0.022 | 0.022 | 0.022 |
| highly susceptible ratio | 0.021 | -0.037 | CVSU | 0.068 | 0.069 | 0.069 | 0.069 | 0.069 |
| terminal room disinfection efficacy | -0.144 | -0.183 | CVSU | >0.1 | >0.1 | >0.1 | >0.1 | >0.1 |
| admission prevalence | 0.935 | 0.909 | CVSU | <0.01 | <0.01 | <0.01 | <0.01 | <0.01 |
| probability env contamination from contaminated hcw | 0.695 | 0.133 | MICU | <0.01 | <0.01 | <0.01 | <0.01 | <0.01 |
| increase factor for highly susceptible | 0.306 | -0.201 | MICU | <0.01 | <0.01 | <0.01 | <0.01 | <0.01 |
| pathogen natural clearance from dry surfaces per day | -0.017 | 0.289 | MICU | <0.01 | <0.01 | <0.01 | <0.01 | <0.01 |
| nurse PPE compliance mean | 0.077 | 0.373 | MICU | <0.01 | <0.01 | <0.01 | <0.01 | <0.01 |
| nurse hygiene compliance exit mean | -0.321 | -0.61 | MICU | <0.01 | <0.01 | <0.01 | <0.01 | <0.01 |
| HCW PPE compliance variability from mean | -0.311 | -0.035 | MICU | <0.01 | <0.01 | <0.01 | <0.01 | <0.01 |
| highly susceptible ratio | 0.172 | 0.04 | MICU | <0.01 | <0.01 | <0.01 | <0.01 | <0.01 |
| probability environmental colonization hourly | 0.147 | 0.02 | MICU | <0.01 | <0.01 | <0.01 | <0.01 | <0.01 |
| HCW hygiene compliance variability from mean | 0.136 | 0.247 | MICU | <0.01 | <0.01 | <0.01 | <0.01 | <0.01 |
| admission prevalence | 0.815 | 0.713 | MICU | <0.01 | <0.01 | <0.01 | <0.01 | <0.01 |
| probability hcw contamination from contaminated env | 0.211 | 0.126 | MICU | <0.01 | <0.01 | <0.01 | <0.01 | <0.01 |
| probability room contamination by colonized patient | -0.186 | -0.266 | MICU | <0.01 | <0.01 | <0.01 | <0.01 | <0.01 |
| test sensitivity | -0.128 | -0.055 | MICU | 0.02 | 0.02 | 0.02 | 0.02 | 0.02 |
| probability HCW contamination from colonized patient | -0.023 | 0.039 | MICU | 0.05 | 0.05 | 0.05 | 0.05 | 0.05 |
| nurse hygiene compliance enter mean | -0.471 | -0.523 | MICU | 0.029 | 0.029 | 0.029 | 0.029 | 0.029 |
| terminal room disinfection efficacy | -0.169 | -0.205 | MICU | >0.1 | >0.1 | >0.1 | >0.1 | >0.1 |
| probability transmission from contaminated hcw to susceptible patient | -0.016 | -0.001 | MICU | >0.1 | >0.1 | >0.1 | >0.1 | >0.1 |
| residual contamination post hand washing | 0.271 | 0.278 | MICU | >0.1 | >0.1 | >0.1 | >0.1 | >0.1 |
| HCW hygiene compliance variability from mean | -0.027 | 0.455 | NCCU | <0.01 | <0.01 | <0.01 | <0.01 | <0.01 |
| probability hcw contamination from contaminated env | -0.041 | 0.367 | NCCU | <0.01 | <0.01 | <0.01 | <0.01 | <0.01 |
| probability env contamination from contaminated hcw | 0.434 | 0.045 | NCCU | <0.01 | <0.01 | <0.01 | <0.01 | <0.01 |
| pathogen natural clearance from dry surfaces per day | 0.09 | 0.404 | NCCU | <0.01 | <0.01 | <0.01 | <0.01 | <0.01 |
| residual contamination post hand washing | 0.195 | -0.097 | NCCU | <0.01 | <0.01 | <0.01 | <0.01 | <0.01 |
| probability environmental colonization hourly | 0.173 | -0.111 | NCCU | <0.01 | <0.01 | <0.01 | <0.01 | <0.01 |
| nurse hygiene compliance exit mean | -0.146 | -0.42 | NCCU | <0.01 | <0.01 | <0.01 | <0.01 | <0.01 |
| HCW PPE compliance variability from mean | -0.237 | 0.002 | NCCU | <0.01 | <0.01 | <0.01 | <0.01 | <0.01 |
| highly susceptible ratio | -0.093 | 0.138 | NCCU | <0.01 | <0.01 | <0.01 | <0.01 | <0.01 |
| probability room contamination by colonized patient | 0.14 | -0.068 | NCCU | <0.01 | <0.01 | <0.01 | <0.01 | <0.01 |
| probability transmission from contaminated hcw to susceptible patient | -0.143 | 0.033 | NCCU | <0.01 | <0.01 | <0.01 | <0.01 | <0.01 |
| terminal room disinfection efficacy | -0.085 | -0.228 | NCCU | <0.01 | <0.01 | <0.01 | <0.01 | <0.01 |
| nurse hygiene compliance enter mean | -0.537 | -0.42 | NCCU | <0.01 | <0.01 | <0.01 | <0.01 | <0.01 |
| test sensitivity | -0.229 | -0.165 | NCCU | 0.033 | 0.033 | 0.033 | 0.033 | 0.033 |
| nurse PPE compliance mean | 0.31 | 0.274 | NCCU | >0.1 | >0.1 | >0.1 | >0.1 | >0.1 |
| admission prevalence | 0.952 | 0.921 | NCCU | <0.01 | <0.01 | <0.01 | <0.01 | <0.01 |
| increase factor for highly susceptible | 0.081 | 0.055 | NCCU | >0.1 | >0.1 | >0.1 | >0.1 | >0.1 |
| probability HCW contamination from colonized patient | -0.071 | -0.067 | NCCU | >0.1 | >0.1 | >0.1 | >0.1 | >0.1 |
| probability env contamination from contaminated hcw | 0.595 | 0.061 | SICU | <0.01 | <0.01 | <0.01 | <0.01 | <0.01 |
| pathogen natural clearance from dry surfaces per day | 0.053 | 0.4 | SICU | <0.01 | <0.01 | <0.01 | <0.01 | <0.01 |
| HCW hygiene compliance variability from mean | 0.084 | 0.414 | SICU | <0.01 | <0.01 | <0.01 | <0.01 | <0.01 |
| HCW PPE compliance variability from mean | -0.272 | -0.001 | SICU | <0.01 | <0.01 | <0.01 | <0.01 | <0.01 |
| nurse hygiene compliance exit mean | -0.27 | -0.511 | SICU | <0.01 | <0.01 | <0.01 | <0.01 | <0.01 |
| increase factor for highly susceptible | 0.109 | -0.13 | SICU | <0.01 | <0.01 | <0.01 | <0.01 | <0.01 |
| probability environmental colonization hourly | 0.158 | -0.074 | SICU | <0.01 | <0.01 | <0.01 | <0.01 | <0.01 |
| probability hcw contamination from contaminated env | 0.107 | 0.292 | SICU | <0.01 | <0.01 | <0.01 | <0.01 | <0.01 |
| probability transmission from contaminated hcw to susceptible patient | -0.116 | 0.068 | SICU | <0.01 | <0.01 | <0.01 | <0.01 | <0.01 |
| probability room contamination by colonized patient | 0.007 | -0.171 | SICU | <0.01 | <0.01 | <0.01 | <0.01 | <0.01 |
| residual contamination post hand washing | 0.323 | 0.175 | SICU | <0.01 | <0.01 | <0.01 | <0.01 | <0.01 |
| nurse hygiene compliance enter mean | -0.569 | -0.453 | SICU | <0.01 | <0.01 | <0.01 | <0.01 | <0.01 |
| nurse PPE compliance mean | 0.216 | 0.311 | SICU | <0.01 | <0.01 | <0.01 | <0.01 | <0.01 |
| highly susceptible ratio | 0.071 | 0.011 | SICU | 0.057 | 0.057 | 0.057 | 0.057 | 0.057 |
| test sensitivity | -0.186 | -0.128 | SICU | 0.057 | 0.058 | 0.058 | 0.058 | 0.058 |
| probability HCW contamination from colonized patient | -0.038 | 0.006 | SICU | >0.1 | >0.1 | >0.1 | >0.1 | >0.1 |
| admission prevalence | 0.922 | 0.884 | SICU | <0.01 | <0.01 | <0.01 | <0.01 | <0.01 |
| terminal room disinfection efficacy | -0.187 | -0.191 | SICU | >0.1 | >0.1 | >0.1 | >0.1 | >0.1 |
| probability env contamination from contaminated hcw | 0.569 | 0.058 | WSICU | <0.01 | <0.01 | <0.01 | <0.01 | <0.01 |
| increase factor for highly susceptible | 0.205 | -0.173 | WSICU | <0.01 | <0.01 | <0.01 | <0.01 | <0.01 |
| HCW PPE compliance variability from mean | -0.297 | 0.02 | WSICU | <0.01 | <0.01 | <0.01 | <0.01 | <0.01 |
| pathogen natural clearance from dry surfaces per day | 0.047 | 0.346 | WSICU | <0.01 | <0.01 | <0.01 | <0.01 | <0.01 |
| HCW hygiene compliance variability from mean | 0.085 | 0.365 | WSICU | <0.01 | <0.01 | <0.01 | <0.01 | <0.01 |
| probability environmental colonization hourly | 0.209 | -0.064 | WSICU | <0.01 | <0.01 | <0.01 | <0.01 | <0.01 |
| probability room contamination by colonized patient | 0.016 | -0.229 | WSICU | <0.01 | <0.01 | <0.01 | <0.01 | <0.01 |
| residual contamination post hand washing | 0.245 | 0.045 | WSICU | <0.01 | <0.01 | <0.01 | <0.01 | <0.01 |
| nurse hygiene compliance exit mean | -0.303 | -0.503 | WSICU | <0.01 | <0.01 | <0.01 | <0.01 | <0.01 |
| probability hcw contamination from contaminated env | 0.107 | 0.274 | WSICU | <0.01 | <0.01 | <0.01 | <0.01 | <0.01 |
| nurse PPE compliance mean | 0.198 | 0.348 | WSICU | <0.01 | <0.01 | <0.01 | <0.01 | <0.01 |
| nurse hygiene compliance enter mean | -0.57 | -0.426 | WSICU | <0.01 | <0.01 | <0.01 | <0.01 | <0.01 |
| probability transmission from contaminated hcw to susceptible patient | -0.055 | 0.037 | WSICU | <0.01 | <0.01 | <0.01 | <0.01 | <0.01 |
| test sensitivity | -0.191 | -0.109 | WSICU | <0.01 | <0.01 | <0.01 | <0.01 | <0.01 |
| highly susceptible ratio | 0.111 | 0.032 | WSICU | 0.012 | 0.012 | 0.012 | 0.012 | 0.012 |
| admission prevalence | 0.904 | 0.859 | WSICU | <0.01 | <0.01 | <0.01 | <0.01 | <0.01 |
| probability HCW contamination from colonized patient | 0.019 | -0.014 | WSICU | >0.1 | >0.1 | >0.1 | >0.1 | >0.1 |
| terminal room disinfection efficacy | -0.231 | -0.233 | WSICU | >0.1 | >0.1 | >0.1 | >0.1 | >0.1 |

Table B10. Sensitivity of distribution boundaries on partial rank correlation coefficients for all model parameters in each ICU under the HEHR scenario (in descending order of significance)

|  | | | | **P values** | | | | |
| --- | --- | --- | --- | --- | --- | --- | --- | --- |
| **Parameter** | **PRCC_1_** | **PRCC_2_** | **ICU** | **Pearson** | **Dunn** | **Steiger** | **Raghunathan** | **Silver** |
| increase factor for highly susceptible | 0.132 | -0.201 | CCU | <0.01 | <0.01 | <0.01 | <0.01 | <0.01 |
| nurse hygiene compliance enter mean | -0.123 | -0.414 | CCU | <0.01 | <0.01 | <0.01 | <0.01 | <0.01 |
| terminal room disinfection efficacy | -0.126 | -0.408 | CCU | <0.01 | <0.01 | <0.01 | <0.01 | <0.01 |
| nurse hygiene compliance exit mean | 0.013 | -0.269 | CCU | <0.01 | <0.01 | <0.01 | <0.01 | <0.01 |
| probability hcw contamination from contaminated env | 0.168 | 0.374 | CCU | <0.01 | <0.01 | <0.01 | <0.01 | <0.01 |
| probability env contamination from contaminated hcw | 0.235 | 0.031 | CCU | <0.01 | <0.01 | <0.01 | <0.01 | <0.01 |
| test sensitivity | -0.137 | 0.029 | CCU | <0.01 | <0.01 | <0.01 | <0.01 | <0.01 |
| probability transmission from contaminated hcw to susceptible patient | -0.124 | 0.001 | CCU | <0.01 | <0.01 | <0.01 | <0.01 | <0.01 |
| HCW PPE compliance variability from mean | -0.07 | 0.045 | CCU | <0.01 | <0.01 | <0.01 | <0.01 | <0.01 |
| HCW hygiene compliance variability from mean | 0.147 | 0.261 | CCU | <0.01 | <0.01 | <0.01 | <0.01 | <0.01 |
| highly susceptible ratio | 0.206 | 0.125 | CCU | <0.01 | <0.01 | <0.01 | <0.01 | <0.01 |
| pathogen natural clearance from dry surfaces per day | 0.174 | 0.105 | CCU | 0.027 | 0.027 | 0.027 | 0.027 | 0.027 |
| admission prevalence | 0.832 | 0.786 | CCU | <0.01 | <0.01 | <0.01 | <0.01 | <0.01 |
| nurse PPE compliance mean | 0.212 | 0.244 | CCU | >0.1 | >0.1 | >0.1 | >0.1 | >0.1 |
| probability HCW contamination from colonized patient | 0.052 | 0.082 | CCU | >0.1 | >0.1 | >0.1 | >0.1 | >0.1 |
| probability environmental colonization hourly | 0.064 | 0.08 | CCU | >0.1 | >0.1 | >0.1 | >0.1 | >0.1 |
| residual contamination post hand washing | -0.126 | -0.132 | CCU | >0.1 | >0.1 | >0.1 | >0.1 | >0.1 |
| probability room contamination by colonized patient | -0.129 | -0.133 | CCU | >0.1 | >0.1 | >0.1 | >0.1 | >0.1 |
| increase factor for highly susceptible | 0.168 | -0.237 | CVSU | <0.01 | <0.01 | <0.01 | <0.01 | <0.01 |
| terminal room disinfection efficacy | -0.102 | -0.403 | CVSU | <0.01 | <0.01 | <0.01 | <0.01 | <0.01 |
| probability hcw contamination from contaminated env | 0.113 | 0.372 | CVSU | <0.01 | <0.01 | <0.01 | <0.01 | <0.01 |
| nurse hygiene compliance enter mean | -0.132 | -0.339 | CVSU | <0.01 | <0.01 | <0.01 | <0.01 | <0.01 |
| nurse hygiene compliance exit mean | -0.038 | -0.234 | CVSU | <0.01 | <0.01 | <0.01 | <0.01 | <0.01 |
| test sensitivity | -0.131 | 0.057 | CVSU | <0.01 | <0.01 | <0.01 | <0.01 | <0.01 |
| probability transmission from contaminated hcw to susceptible patient | -0.179 | 0.007 | CVSU | <0.01 | <0.01 | <0.01 | <0.01 | <0.01 |
| probability env contamination from contaminated hcw | 0.162 | -0.004 | CVSU | <0.01 | <0.01 | <0.01 | <0.01 | <0.01 |
| HCW hygiene compliance variability from mean | 0.108 | 0.262 | CVSU | <0.01 | <0.01 | <0.01 | <0.01 | <0.01 |
| HCW PPE compliance variability from mean | -0.065 | 0.072 | CVSU | <0.01 | <0.01 | <0.01 | <0.01 | <0.01 |
| probability HCW contamination from colonized patient | -0.029 | 0.085 | CVSU | <0.01 | <0.01 | <0.01 | <0.01 | <0.01 |
| pathogen natural clearance from dry surfaces per day | 0.183 | 0.089 | CVSU | <0.01 | <0.01 | <0.01 | <0.01 | <0.01 |
| highly susceptible ratio | 0.159 | 0.093 | CVSU | 0.036 | 0.036 | 0.036 | 0.036 | 0.036 |
| probability environmental colonization hourly | 0.032 | -0.029 | CVSU | 0.055 | 0.055 | 0.055 | 0.055 | 0.055 |
| probability room contamination by colonized patient | -0.109 | -0.147 | CVSU | >0.1 | >0.1 | >0.1 | >0.1 | >0.1 |
| residual contamination post hand washing | -0.099 | -0.128 | CVSU | >0.1 | >0.1 | >0.1 | >0.1 | >0.1 |
| admission prevalence | 0.856 | 0.829 | CVSU | <0.01 | <0.01 | <0.01 | <0.01 | <0.01 |
| nurse PPE compliance mean | 0.254 | 0.276 | CVSU | >0.1 | >0.1 | >0.1 | >0.1 | >0.1 |
| increase factor for highly susceptible | 0.153 | -0.2 | MICU | <0.01 | <0.01 | <0.01 | <0.01 | <0.01 |
| terminal room disinfection efficacy | -0.12 | -0.463 | MICU | <0.01 | <0.01 | <0.01 | <0.01 | <0.01 |
| nurse hygiene compliance enter mean | -0.056 | -0.356 | MICU | <0.01 | <0.01 | <0.01 | <0.01 | <0.01 |
| nurse hygiene compliance exit mean | 0.041 | -0.25 | MICU | <0.01 | <0.01 | <0.01 | <0.01 | <0.01 |
| probability transmission from contaminated hcw to susceptible patient | -0.212 | -0.006 | MICU | <0.01 | <0.01 | <0.01 | <0.01 | <0.01 |
| highly susceptible ratio | 0.285 | 0.112 | MICU | <0.01 | <0.01 | <0.01 | <0.01 | <0.01 |
| pathogen natural clearance from dry surfaces per day | 0.212 | 0.04 | MICU | <0.01 | <0.01 | <0.01 | <0.01 | <0.01 |
| probability env contamination from contaminated hcw | 0.186 | 0.03 | MICU | <0.01 | <0.01 | <0.01 | <0.01 | <0.01 |
| probability hcw contamination from contaminated env | 0.186 | 0.339 | MICU | <0.01 | <0.01 | <0.01 | <0.01 | <0.01 |
| test sensitivity | -0.123 | 0.03 | MICU | <0.01 | <0.01 | <0.01 | <0.01 | <0.01 |
| probability HCW contamination from colonized patient | -0.055 | 0.035 | MICU | <0.01 | <0.01 | <0.01 | <0.01 | <0.01 |
| probability environmental colonization hourly | -0.006 | 0.081 | MICU | <0.01 | <0.01 | <0.01 | <0.01 | <0.01 |
| nurse PPE compliance mean | 0.187 | 0.27 | MICU | <0.01 | <0.01 | <0.01 | <0.01 | <0.01 |
| HCW hygiene compliance variability from mean | 0.113 | 0.189 | MICU | 0.014 | 0.014 | 0.014 | 0.014 | 0.014 |
| admission prevalence | 0.687 | 0.617 | MICU | <0.01 | <0.01 | <0.01 | <0.01 | <0.01 |
| HCW PPE compliance variability from mean | -0.01 | 0.039 | MICU | >0.1 | >0.1 | >0.1 | >0.1 | >0.1 |
| residual contamination post hand washing | -0.152 | -0.143 | MICU | >0.1 | >0.1 | >0.1 | >0.1 | >0.1 |
| probability room contamination by colonized patient | -0.157 | -0.16 | MICU | >0.1 | >0.1 | >0.1 | >0.1 | >0.1 |
| increase factor for highly susceptible | 0.128 | -0.2 | NCCU | <0.01 | <0.01 | <0.01 | <0.01 | <0.01 |
| probability hcw contamination from contaminated env | 0.11 | 0.408 | NCCU | <0.01 | <0.01 | <0.01 | <0.01 | <0.01 |
| terminal room disinfection efficacy | -0.166 | -0.433 | NCCU | <0.01 | <0.01 | <0.01 | <0.01 | <0.01 |
| probability transmission from contaminated hcw to susceptible patient | -0.193 | 0.072 | NCCU | <0.01 | <0.01 | <0.01 | <0.01 | <0.01 |
| probability env contamination from contaminated hcw | 0.227 | -0.003 | NCCU | <0.01 | <0.01 | <0.01 | <0.01 | <0.01 |
| nurse hygiene compliance enter mean | -0.226 | -0.432 | NCCU | <0.01 | <0.01 | <0.01 | <0.01 | <0.01 |
| nurse hygiene compliance exit mean | -0.063 | -0.262 | NCCU | <0.01 | <0.01 | <0.01 | <0.01 | <0.01 |
| HCW hygiene compliance variability from mean | 0.115 | 0.296 | NCCU | <0.01 | <0.01 | <0.01 | <0.01 | <0.01 |
| test sensitivity | -0.146 | -0.006 | NCCU | <0.01 | <0.01 | <0.01 | <0.01 | <0.01 |
| residual contamination post hand washing | -0.081 | -0.185 | NCCU | <0.01 | <0.01 | <0.01 | <0.01 | <0.01 |
| pathogen natural clearance from dry surfaces per day | 0.209 | 0.108 | NCCU | <0.01 | <0.01 | <0.01 | <0.01 | <0.01 |
| HCW PPE compliance variability from mean | -0.053 | 0.025 | NCCU | 0.013 | 0.013 | 0.013 | 0.013 | 0.013 |
| highly susceptible ratio | 0.158 | 0.097 | NCCU | 0.052 | 0.052 | 0.052 | 0.052 | 0.052 |
| probability HCW contamination from colonized patient | 0.032 | 0.081 | NCCU | >0.1 | >0.1 | >0.1 | >0.1 | >0.1 |
| probability room contamination by colonized patient | -0.117 | -0.154 | NCCU | >0.1 | >0.1 | >0.1 | >0.1 | >0.1 |
| probability environmental colonization hourly | 0.006 | -0.03 | NCCU | >0.1 | >0.1 | >0.1 | >0.1 | >0.1 |
| admission prevalence | 0.918 | 0.885 | NCCU | <0.01 | <0.01 | <0.01 | <0.01 | <0.01 |
| nurse PPE compliance mean | 0.241 | 0.272 | NCCU | >0.1 | >0.1 | >0.1 | >0.1 | >0.1 |
| increase factor for highly susceptible | 0.173 | -0.185 | SICU | <0.01 | <0.01 | <0.01 | <0.01 | <0.01 |
| nurse hygiene compliance exit mean | 0.044 | -0.266 | SICU | <0.01 | <0.01 | <0.01 | <0.01 | <0.01 |
| terminal room disinfection efficacy | -0.169 | -0.449 | SICU | <0.01 | <0.01 | <0.01 | <0.01 | <0.01 |
| probability hcw contamination from contaminated env | 0.125 | 0.385 | SICU | <0.01 | <0.01 | <0.01 | <0.01 | <0.01 |
| nurse hygiene compliance enter mean | -0.166 | -0.389 | SICU | <0.01 | <0.01 | <0.01 | <0.01 | <0.01 |
| probability transmission from contaminated hcw to susceptible patient | -0.182 | -0.006 | SICU | <0.01 | <0.01 | <0.01 | <0.01 | <0.01 |
| HCW hygiene compliance variability from mean | 0.075 | 0.234 | SICU | <0.01 | <0.01 | <0.01 | <0.01 | <0.01 |
| pathogen natural clearance from dry surfaces per day | 0.192 | 0.046 | SICU | <0.01 | <0.01 | <0.01 | <0.01 | <0.01 |
| test sensitivity | -0.158 | -0.013 | SICU | <0.01 | <0.01 | <0.01 | <0.01 | <0.01 |
| probability environmental colonization hourly | -0.008 | 0.119 | SICU | <0.01 | <0.01 | <0.01 | <0.01 | <0.01 |
| probability env contamination from contaminated hcw | 0.195 | 0.084 | SICU | <0.01 | <0.01 | <0.01 | <0.01 | <0.01 |
| residual contamination post hand washing | -0.103 | -0.209 | SICU | <0.01 | <0.01 | <0.01 | <0.01 | <0.01 |
| probability room contamination by colonized patient | -0.051 | -0.149 | SICU | <0.01 | <0.01 | <0.01 | <0.01 | <0.01 |
| highly susceptible ratio | 0.194 | 0.111 | SICU | <0.01 | <0.01 | <0.01 | <0.01 | <0.01 |
| HCW PPE compliance variability from mean | -0.057 | 0.025 | SICU | 0.01 | 0.01 | 0.01 | 0.01 | 0.01 |
| nurse PPE compliance mean | 0.228 | 0.297 | SICU | 0.018 | 0.018 | 0.018 | 0.018 | 0.018 |
| probability HCW contamination from colonized patient | -0.018 | 0.038 | SICU | 0.075 | 0.075 | 0.075 | 0.075 | 0.075 |
| admission prevalence | 0.821 | 0.778 | SICU | <0.01 | <0.01 | <0.01 | <0.01 | <0.01 |
| increase factor for highly susceptible | 0.207 | -0.182 | WSICU | <0.01 | <0.01 | <0.01 | <0.01 | <0.01 |
| probability env contamination from contaminated hcw | 0.285 | 0.002 | WSICU | <0.01 | <0.01 | <0.01 | <0.01 | <0.01 |
| terminal room disinfection efficacy | -0.199 | -0.472 | WSICU | <0.01 | <0.01 | <0.01 | <0.01 | <0.01 |
| nurse hygiene compliance enter mean | -0.15 | -0.391 | WSICU | <0.01 | <0.01 | <0.01 | <0.01 | <0.01 |
| nurse hygiene compliance exit mean | -0.053 | -0.27 | WSICU | <0.01 | <0.01 | <0.01 | <0.01 | <0.01 |
| probability transmission from contaminated hcw to susceptible patient | -0.181 | 0.015 | WSICU | <0.01 | <0.01 | <0.01 | <0.01 | <0.01 |
| probability hcw contamination from contaminated env | 0.147 | 0.304 | WSICU | <0.01 | <0.01 | <0.01 | <0.01 | <0.01 |
| HCW PPE compliance variability from mean | -0.083 | 0.057 | WSICU | <0.01 | <0.01 | <0.01 | <0.01 | <0.01 |
| highly susceptible ratio | 0.252 | 0.113 | WSICU | <0.01 | <0.01 | <0.01 | <0.01 | <0.01 |
| pathogen natural clearance from dry surfaces per day | 0.186 | 0.053 | WSICU | <0.01 | <0.01 | <0.01 | <0.01 | <0.01 |
| nurse PPE compliance mean | 0.181 | 0.311 | WSICU | <0.01 | <0.01 | <0.01 | <0.01 | <0.01 |
| test sensitivity | -0.131 | -0.028 | WSICU | <0.01 | <0.01 | <0.01 | <0.01 | <0.01 |
| HCW hygiene compliance variability from mean | 0.174 | 0.264 | WSICU | <0.01 | <0.01 | <0.01 | <0.01 | <0.01 |
| admission prevalence | 0.843 | 0.776 | WSICU | <0.01 | <0.01 | <0.01 | <0.01 | <0.01 |
| probability environmental colonization hourly | 0.033 | 0.075 | WSICU | >0.1 | >0.1 | >0.1 | >0.1 | >0.1 |
| residual contamination post hand washing | -0.086 | -0.104 | WSICU | >0.1 | >0.1 | >0.1 | >0.1 | >0.1 |
| probability room contamination by colonized patient | -0.204 | -0.188 | WSICU | >0.1 | >0.1 | >0.1 | >0.1 | >0.1 |
| probability HCW contamination from colonized patient | 0.039 | 0.041 | WSICU | >0.1 | >0.1 | >0.1 | >0.1 | >0.1 |

**B3.4. Results of intervention simulations**


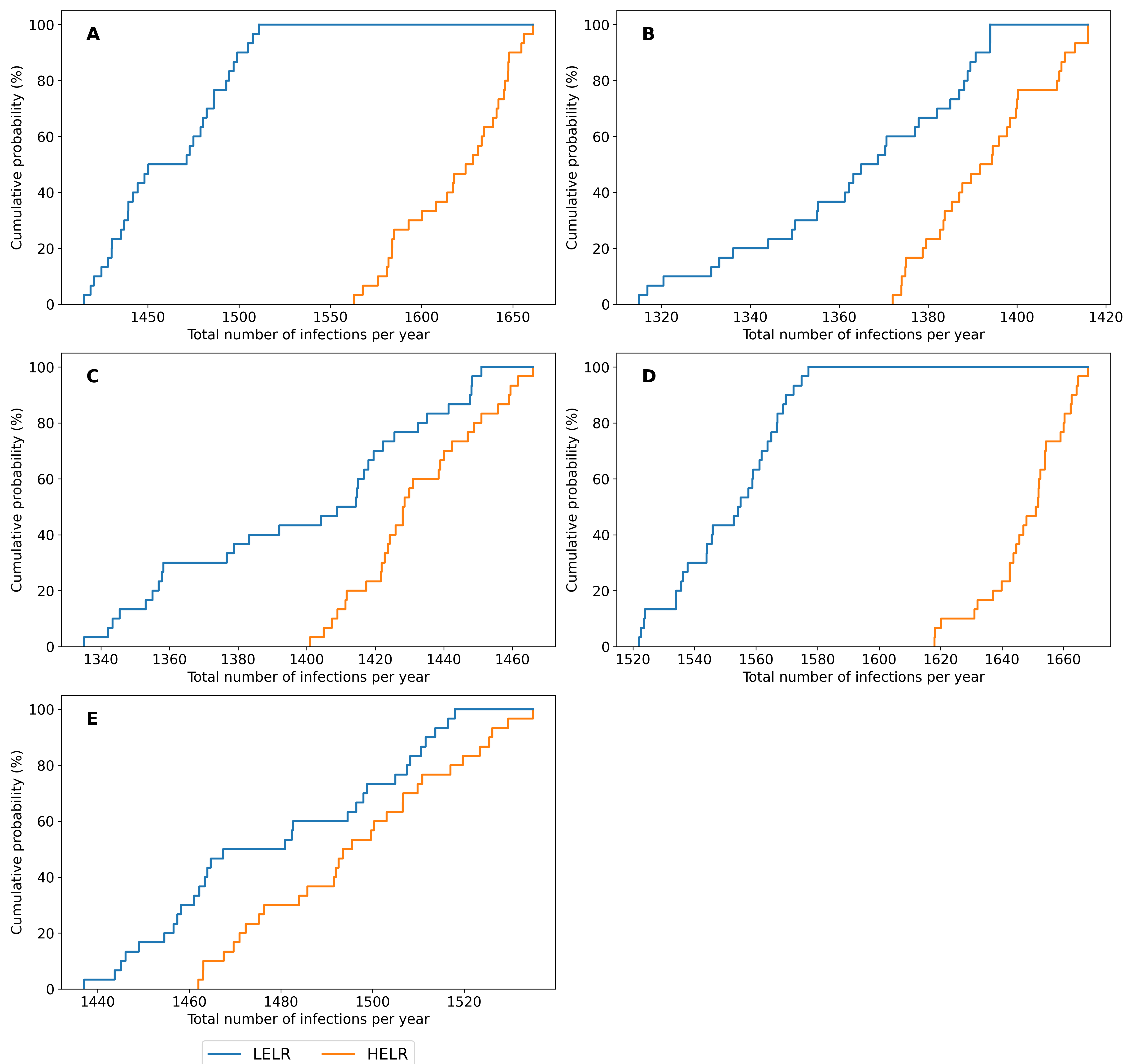


Figure B4. Cumulative probability plot (cumulative distribution function or CDF) for the annual number of infections after the following intervention: improving least hygiene compliance acceptable threshold to 75% (**A**), improving mean HCW hygiene compliance probability on entry to 95% (**B**), improving mean HCW hygiene compliance probability on exit to 95% (**C**), reducing post-hand-washing residual contamination to 1% (**D**), and improving terminal room disinfection efficacy to 95% (**E**). Dashed lines show the 50^th^ and 95^th^ percentiles. LELR: low probability of direct environmental colonization and low levels of residual contamination; HELR: high probability of direct environmental colonization and low levels of residual contamination.

**References**

1. Marino S, Hogue IB, Ray CJ, Kirschner DE. A methodology for performing global uncertainty and sensitivity analysis in systems biology. J Theor Biol **2008**; 254:178–196.
